# *Dracocephalum moldavica* and *Nepeta racemosa* Essential Oils: A Review of Chemical Composition, Biological Activities, and Anti-*Helicobacter pylori* Potential

**DOI:** 10.3390/molecules31203599

**Published:** 2026-10-10

**Authors:** Oana M. Grigor, Carmen R. Pop, Alina L. Nistor, Rodica Vârban, Ileana C. Nicoară, Liana C. Salanţă, Ancuța M. Rotar

**Affiliations:** 1 Department of Food Science, Faculty of Food Science and Technology, University of Agricultural Science and Veterinary Medicine, 400372 Cluj-Napoca, Romania; oana-maria.grigor@usamvcluj.ro (O.M.G.); alina.nistor@usamvcluj.ro (A.L.N.); liana.salanta@usamvcluj.ro (L.C.S.); anca.rotar@usamvcluj.ro (A.M.R.); 2 Department of Crop Science, Faculty of Agriculture, University of Agricultural Science and Veterinary Medicine, 400372 Cluj-Napoca, Romania; rodica.varban@usamvcluj.ro; 3 Department of Rheumatology, “Iuliu Hațieganu” University of Medicine and Pharmacy, Cluj Clinical County Emergency Hospital, 400012 Cluj-Napoca, Romania; ileana_nicoara@yahoo.com

**Keywords:** *Dracocephalum moldavica*, *Nepeta racemosa*, essential oil, antibacterial activity, *Helicobacter pylori*

## Abstract

*Dracocephalum moldavica* and *Nepeta racemosa* essential oils (DMEO and NREO) are under-studied *Lamiaceae* oils whose major constituents—citral-type monoterpenes and nepetalactones, respectively—have been independently associated with antibacterial and anti-*Helicobacter pylori* activity; however, the literature on both oils remains fragmented. This comprehensive narrative review, based on a structured search of Web of Science, Scopus, PubMed, and Google Scholar (2016–2026), integrates 78 publications on their chemical composition, antioxidant and antibacterial activities, mechanisms of action, applications, and toxicological profiles. DMEO is dominated by oxygenated monoterpenes, chiefly geranyl acetate, geranial, neral, and geraniol, and exhibits broad-spectrum antibacterial activity, generally stronger against Gram-positive bacteria, whereas its antioxidant activity is weaker than that of synthetic standards. NREO composition is markedly chemotype-dependent, ranging from nepetalactone-dominant to 1,8-cineole- or citral-rich profiles, and direct bioactivity and toxicity data remain scarce. Overall, DMEO has a considerably stronger evidence base than NREO, and safety conclusions for both oils rely largely on data for major constituents rather than on whole-oil testing. The main limitations, namely methodological heterogeneity and the absence of direct anti-*H. pylori* data, define the research priorities: MIC/MBC testing of chemically characterized oils against reference and clinical *H. pylori* strains, antibiotic synergy, gastric cytotoxicity, and in vivo validation.

## 1. Introduction

Essential oils (EOs) are volatile, aromatic mixtures biosynthesized by plants and widely utilized in the pharmaceutical, food, cosmetic, and fragrance industries [1]. They typically comprise between 20 and 60 constituents and are generally dominated by two or three major components present at concentrations of 20–70%, which primarily dictate their biological efficacy [2]. These constituents are predominantly terpenes and their oxygenated derivatives, terpenoids, which exhibit antimicrobial, antioxidant, anti-inflammatory, and anticancer activities [3].

Only approximately 10% of plant species are aromatic and capable of producing EOs, particularly within families such as *Lamiaceae*, *Apiaceae*, *Rutaceae*, *Myrtaceae*, *Lauraceae*, and *Asteraceae* [4]. These aromatic species represent a subgroup of medicinal and aromatic plants (MAPs), characterized by their ability to synthesize bioactive compounds with therapeutic and sensory properties [5]. MAPs are used across diverse sectors, including pharmaceuticals, food production, agriculture, and cosmetics, largely owing to their sustainability and reduced environmental impact compared to synthetic counterparts [6], and are also distributed across numerous other botanical families [7]. Among these EO-yielding families, *Lamiaceae* comprises over 7000 species across approximately 240–250 genera [8] and represents an important source of EOs with sensory and therapeutic value [9]. *Dracocephalum moldavica*, commonly referred to as Moldavian dragonhead or Moldavian balm, is an annual aromatic herb native to temperate regions of Asia, with subsequent distribution across Europe, China, and the northeastern United States [10]. The species has been widely integrated into traditional medicinal systems, particularly in Iran, where it has been employed as a reconstituting, cardiotonic, and diaphoretic agent, as well as for the management of gastrointestinal disturbances, insect stings, and snake bites. Beyond its ethnomedical relevance, *Dracocephalum moldavica* is extensively utilized in the food and cosmetic industries as a flavoring agent in teas, infusions, and alcoholic beverages, as well as in perfumery.

*Dracocephalum moldavica* essential oil (DMEO), obtained from the flowering aerial parts of the plant, is predominantly composed of oxygenated acyclic monoterpenes such as geranyl acetate, geranial, geraniol, neral, and neryl acetate, which are responsible for its characteristic citrus-like aroma and are closely associated with its documented antimicrobial activity against pathogenic microorganisms including *Staphylococcus aureus*, *Escherichia coli*, *Salmonella* Typhimurium, and *Listeria monocytogenes*, an efficacy further correlated with its antioxidant capacity [11,12].

According to Abbasi et al. [13], DMEO yield and chemical composition vary significantly depending on the phenological stage of *D. moldavica*, with maximum yields (0.50–0.88% *v*/*w*) recorded during the flowering phase. At the compositional level, notable variations in oxygenated monoterpenes have been observed: geranyl acetate predominates during the floral budding stage, whereas geranial and citronellal are more abundant during flowering and fruit set. These changes are associated with the regulated expression of the geraniol synthase gene, as well as reversible acetylation and deacetylation processes that govern the interconversion between geraniol and geranyl acetate throughout inflorescence development.

The genus *Nepeta* (family *Lamiaceae*) comprises approximately 300 species characterized by considerable phenotypic plasticity and a primary distribution in Southwest Asia. Among these, *Nepeta racemosa*, with synonyms including *Nepeta elbursensis* Rech. f., *Nepeta mussinii* Spreng., *Nepeta grandiflora* M.B., and *Nepeta nepetella* subsp. *marifolia* (Boiss. & Huet), is particularly notable for its high content of nepetalactones, a class of iridoid monoterpenes characteristic of *Nepeta racemosa* essential oil (NREO). Compounds of this class are traditionally associated with antiseptic, astringent, antiasthmatic, sedative, antitussive, expectorant, antispasmodic, antimicrobial, antiviral, antifungal, and insecticidal properties, alongside bioherbicidal and pheromonal activity relevant to plant protection strategies, particularly in relation to aphid behavior [14,15,16,17,18]. Beyond its medicinal relevance, *N. racemosa* has historically been employed as a culinary ingredient since the Roman period, incorporated into regional dishes such as soups in certain areas of present-day Turkey, and has been used in the management of a wide range of conditions, including envenomation, oral and hepatic disorders, renal dysfunction, and cardiovascular diseases such as angina pectoris, tachycardia, and cardiac thrombosis [15,16,19].

NREO incorporated into polymeric scaffolds has shown antibacterial activity against pathogenic microorganisms, such as *S. aureus* and *E. coli*, associated with structural alterations in bacterial cells including membrane disruption, cellular lysis, and agglutination [20].

*D. moldavica* and *N. racemosa* were selected for joint analysis not on the basis of chemical similarity alone, since NREO displays marked chemotypic variability, but because they represent two contrasting yet complementary cases within the *Lamiaceae* family. DMEO is dominated by acyclic oxygenated monoterpenes such as geranial, neral, and geraniol, whereas NREO is typically characterized by iridoid monoterpenes of the nepetalactone type. Constituents from both chemical classes have been independently associated with antibacterial and anti-*Helicobacter pylori* activity, yet direct evidence for the activity of either oil against this pathogen remains insufficient. Moreover, the two oils differ considerably in the extent of the available evidence, DMEO being comparatively well documented, whereas direct biological data for NREO remain scarce. Their joint analysis therefore allows the evidence available for a better-characterized oil to be contrasted with that of a poorly characterized one, highlighting the specific gaps that must be addressed before either oil can be considered for food-related or anti-*H. pylori* applications.

**Figure 1:**
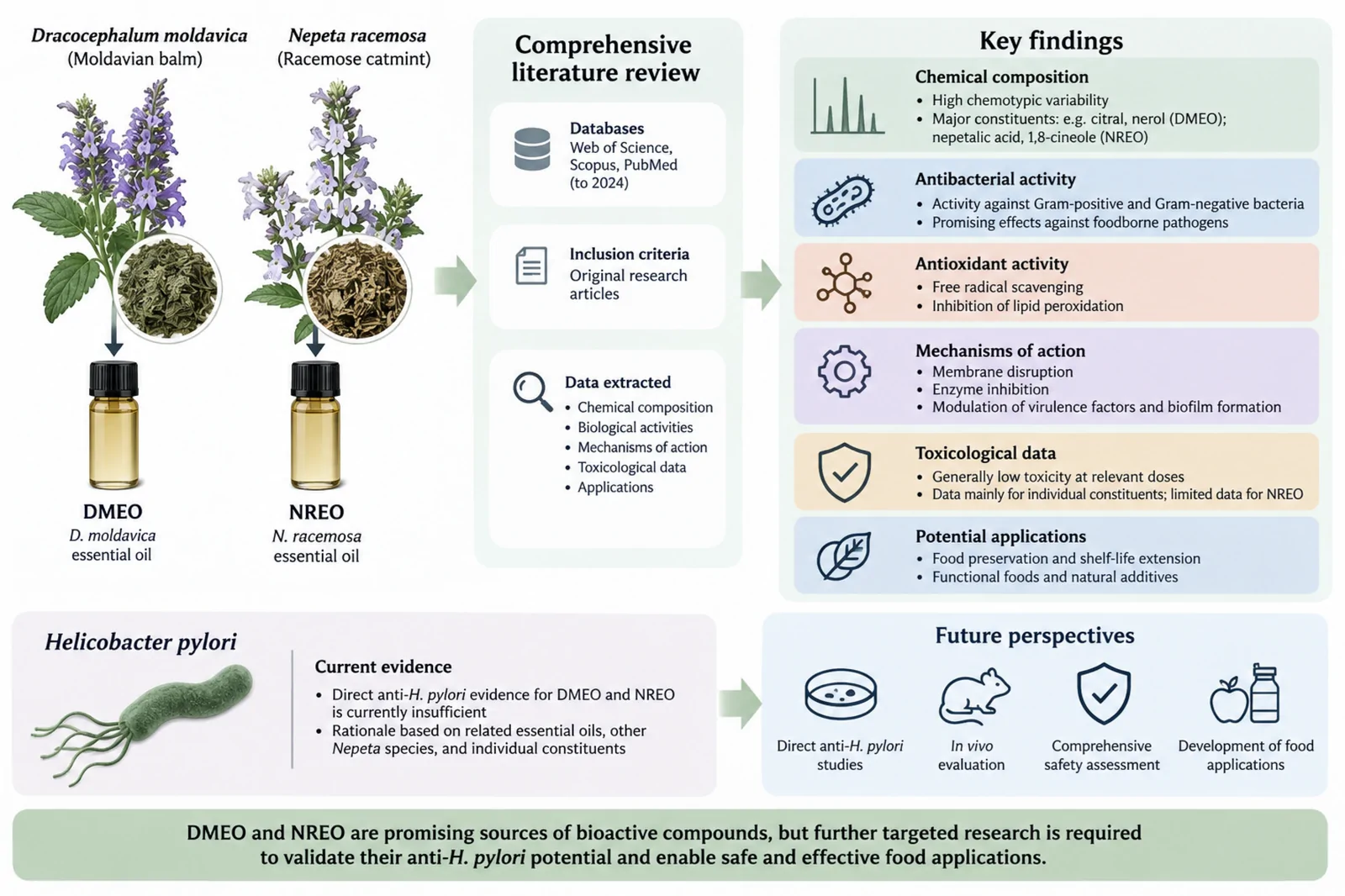
Overview of the chemical composition, biological activities, applications, and research perspectives of DMEO and NREO. The figure was generated with the assistance of ChatGPT (OpenAI, San Francisco, CA, USA; GPT-5.6 Luna) based on the content of the manuscript and the authors’ specifications. The authors reviewed and edited the generated output and take full responsibility for the scientific content and accuracy of the figure.

Despite the growing interest in *Lamiaceae* EOs, the available literature on *D. moldavica* and *N. racemosa* remains fragmented, and information from related *Nepeta* species or individual constituents cannot be directly extrapolated to *N. racemosa*. Therefore, this review aims to (i) compare the chemical composition and chemotypic variability of DMEO and NREO; (ii) critically evaluate their antioxidant and antibacterial activities; (iii) discuss the proposed mechanisms of action of the oils and their major constituents; (iv) summarize their demonstrated and proposed applications, particularly in food systems; (v) assess the available toxicological and safety data; and (vi) evaluate the current evidence regarding their potential activity against *H. pylori* and define priorities for future research (Figure 1).

## 2. Methodology of the Narrative Review

The present work was designed as a comprehensive narrative review based on a structured literature search. The data for DMEO and NREO were extracted from scientific databases and analyzed in order to present comprehensive insights into their volatile compositions, biological activities, mechanisms of action, food matrix applications, toxicological profiles, and potential as anti-*Helicobacter pylori* agents. Web of Science, Scopus, PubMed, and Google Scholar databases were used to search peer-reviewed publications written in English. The search covered the period between 2016 and 2026 in order to focus on the most recent evidence available for DMEO and NREO, while earlier findings on their chemical composition and biological activities were considered through previously published reviews included in the present work. The keywords used in the search were: essential oils, *Dracocephalum moldavica* L., *Nepeta racemosa* Lam., chemical composition, GC-MS, antioxidant activity, antibacterial activity, mechanism of action, food matrix, and anti-*Helicobacter pylori* activity, alone or in various combinations.

Studies were included if they: (i) were peer-reviewed original research articles, reviews, or book chapters published in English; (ii) investigated DMEO or NREO obtained through various extraction methods and reported data on their chemical composition by GC-MS, antioxidant or antibacterial activity, mechanisms of action, food and other applications, including encapsulation or delivery systems, or toxicological profiles; (iii) investigated the major constituents of DMEO and NREO, or EOs from related *Nepeta* species, in cases where direct data for DMEO and NREO were unavailable; (iv) investigated the activity of EOs or their constituents against *H. pylori*; or (v) were regulatory documents, official scientific opinions, or toxicological database entries (e.g., PubChem, ECHA) relevant to the safety assessment of DMEO and NREO constituents, irrespective of their publication date.

Studies were excluded if they: (i) investigated non-volatile plant extracts, hydrolates, or extract fractions of *D. moldavica* or *N. racemosa* rather than DMEO or NREO; (ii) did not clearly present the methods or results; or (iii) were conference abstracts, editorials, or other non-peer-reviewed publications, with the exception of the regulatory documents and database entries mentioned above. According to these criteria, 78 publications were included in the current comprehensive narrative review.

## 3. Chemical Composition of DMEO and NREO

Numerous species within the *Lamiaceae* family are highly valued for their aromatic characteristics and the production of essential oils, which underpin their widespread applications in culinary, medicinal, and ornamental contexts [9]. In this regard, the following section focuses on the identification and characterization of the major volatile compounds present in DMEO and NREO.

### 3.1. Chemical Composition of DMEO

GC–MS analyses of DMEO indicate that approximately 98.0–98.5% of the volatile fraction has been identified, with the number of detected compounds ranging between 15 and 28. The chemical profile is dominated by oxygenated monoterpenes, which account for approximately 85.4–95.1% of the total composition, while monoterpene hydrocarbons are present in lower proportions (approximately 4.0–4.3%), followed by trace levels of sesquiterpene hydrocarbons (≤0.2%) and other minor constituents (approximately 1.9%) [11,21,22].

The principal constituents identified across studies include geranyl acetate, geranial, neral, geraniol, and linalool, with notable quantitative variability. Geranyl acetate is frequently the dominant compound, ranging from approximately 16.7% to 53.2%, followed by geranial (≈9.3–33.9%) and neral (≈7.6–22.7%), while geraniol ranges between approximately 1.3% and 24.3%. Linalool is generally present in smaller amounts (≈0.8–4.1%). Minor constituents, each typically below 5%, are summarized in Table 1 [11,21,22,23,24,25].

A comparative overview of these compositional profiles is provided in Table 1, in which DMEO constituents are expressed as relative percentages (% of total identified compounds). Despite the consistent identification of the key compounds across studies conducted in different regions, notable variations in relative composition are reported, which are commonly associated with factors such as plant origin, genetic and physiological characteristics, environmental and climatic conditions, harvest period, agronomic practices, and distillation techniques [22].

A direct comparison of the individual profiles summarized in Table 1 allows two main DMEO chemotypes to be distinguished. The first, and most frequently reported, is a geranyl acetate chemotype, in which geranyl acetate is the single most abundant constituent (32.57–53.2%) [11,21,23,24]. Within this group, two variants can be recognized: a geranyl acetate/citral variant, in which geranial and neral are the next most abundant constituents (jointly 27.5–47.15%), while geraniol is either absent or present at lower levels (≤8.78%) [21,23], and a geranyl acetate/geraniol variant, in which geraniol reaches 20.2–24.31% and exceeds each of the two citral isomers [11,24]. The second is a citral chemotype, in which the combined content of geranial and neral (49.73–56.08%) exceeds that of geranyl acetate (16.72–19.27%) [22,25]. Since these chemotypes share the same major constituents and differ mainly in their relative proportions, they reflect quantitative rather than qualitative differences in composition, consistent with the phenology-dependent interconversion between geraniol and geranyl acetate reported by Abbasi et al. [13].

### 3.2. Chemical Composition of NREO

During the early flowering stage (first harvest, early May), NREO yield has been reported at 0.35 ± 0.08%, with GC–MS analysis identifying nepetalic acid as the predominant component (55.5%), followed by 1,8-cineole (10.7%) and 3-hydroxy-dihydronepetalactone (5.4%), while minor constituents were present in substantially lower proportions [20]. In a more comprehensive compositional analysis, a total of 53 compounds were identified, accounting for 77.1% of the total EO, with dominant constituents including 4aα,7α,7aα-nepetalactone (31.70%), germacrene D (7.39%), and n-hexadecanoic acid (6.47%), distributed across several chemical classes led by oxygenated monoterpenes, followed by sesquiterpene hydrocarbons, oxygenated sesquiterpenes, fatty acid derivatives, and minor amounts of monoterpene hydrocarbons and non-terpenoid compounds such as ketones and aldehydes [26].

Based on the high nepetalactone content reported in this analysis, this NREO sample can be classified within the group of *Nepeta* EOs in which nepetalactones constitute the principal volatile components, in contrast to profiles lacking nepetalactones, which are typically characterized by higher levels of compounds such as 1,8-cineole and caryophyllene oxide, detected only in low concentrations in this case. Among nepetalactone stereoisomers, 4aα,7α,7aα-nepetalactone is frequently reported as the predominant form, also described as a major constituent in the EOs of other *Nepeta* species, including *N. cadmea*, *N. racemosa*, *N. cephalotes*, *N. govaniana*, and *N. teydea* [26].

Table 2 provides a comparative summary of these compositional profiles across the studies reviewed, with NREO constituents expressed as relative percentages (% of total identified compounds).

## 4. Biological Properties of *Dracocephalum moldavica* L. and *Nepeta racemosa* Lam. EOs

The biological activities summarized in this section were assessed using different methodologies, including different assay types, EO concentrations, solvents, inoculum sizes, reference compounds, microbial strains, and units of expression. Therefore, the reported values are discussed primarily within each study, and direct quantitative comparisons across studies should be interpreted with caution.

### 4.1. Antioxidant Activity

The antioxidant potential of DMEO is one of its most extensively studied biological properties and is strongly associated with its high geranyl acetate content. At a concentration of 250 mg/mL, DMEO exhibited scavenging activity against lipid radicals (397.20 ± 36.12 µmol TE/100 mL), ABTS•^+^ (312.54 ± 11.63 µmol TE/100 mL), superoxide anions (294.77 ± 13.29 µmol TE/100 mL), and DPPH• radicals (246.39 ± 1.17 µmol TE/100 mL), together with reducing power (171.46 ± 2.56 µmol TE/100 mL) [23]. These values, obtained at a high EO concentration, indicate measurable radical-scavenging and reducing capacity; however, when compared with synthetic standards, the antioxidant activity of DMEO appears considerably weaker [22].

Direct antioxidant activity data specific to NREO itself remain limited in the available literature, the antioxidant assessments reported for *N. racemosa* have instead targeted polar extracts and their phenolic-rich fractions rather than the EO fraction, indicating a gap in the current understanding of the antioxidant potential of NREO as a volatile oil.

The compositional inconsistency noted above is, in fact, characteristic of *N. racemosa* across the wider literature. A comparative synthesis of interspecific germplasm studies compiled by Gomes et al. [31] shows that reported NREO profiles range from a 1,8-cineole-dominated chemotype (51.2%, with essentially no nepetalactone detected) in one population to nepetalactone contents as low as 31.5% and as high as 91.5% in others, consistent with the 91.0% value for the 4aα,7α,7aβ-nepetalactone isomer reported by Azizian et al. [29]. This places *N. racemosa* at the intersection of the two principal EO chemotypes recognized across the genus *Nepeta*, a nepetalactone-dominant chemotype and a 1,8-cineole/lemon-scented (citral-type) chemotype, the latter also encompassing compounds such as neral, geranial, citronellol and geraniol [31], rather than belonging unambiguously to either. This marked chemotypic plasticity, likely influenced by genotype, geographic origin and cultivation conditions, offers a plausible explanation for the scarcity and inconsistency of the bioactivity data available for NREO. The antioxidant implications of this chemotypic divide are examined below, in relation to nepetalactone-dominant Nepeta EOs more broadly.

Sheykhjan et al. [22] characterized both the neat EO (DMEO) and a Tween 80-based nano-emulsion (DMNEO) obtained from Iranian-grown *D. moldavica*. DMEO contained geranial (33.94%), neral (22.4%) and geranyl acetate (19.27%) as major constituents (jointly 75.61% of the oil) and displayed a total phenolic content of 52.79 mg gallic acid equivalents (GAE)/g dry plant material and a total flavonoid content of 43.2 mg quercetin equivalents (QE)/g dry plant material. Using DPPH and ABTS radical-scavenging assays, antioxidant potency ranked BHT > DMEO > DMEO10% > DMNEO on a product-concentration (IC_50_) basis, at equivalent EO content, however, the nano-emulsified form (DMNEO) showed enhanced antioxidant activity relative to the free oil, attributed to the greater aqueous dispersibility and surface area of the nano-droplets. These results are consistent with earlier work on Egyptian-grown *D. moldavica* EO, which reported a considerably weaker DPPH radical-scavenging capacity for the neat EO (IC_50_ = 410 mg/mL) than for the synthetic standard BHT (IC_50_ = 3.18 mg/mL), attributed to constituents such as neral, geraniol, α-terpinene, eugenol and thymol [22].

Within the nepetalactone-chemotype cluster of the genus, *N. sintenisii* Bornm. EO showed weak antioxidant activity in both DPPH and FRAP assays (IC_50_ = 7.16 mg/mL and 0.82 mM Fe^2+^/mg EO, respectively) [32,33], while *N. flavida* Hub.-Mor.EO produced a DPPH IC_50_ of 42.8 ± 2.2 µg/mL [15]. These values indicate that nepetalactone-dominant *Nepeta* EOs generally display modest antioxidant activity, a pattern that, if it extends to *N. racemosa*, would be consistent with the compositional dominance of nepetalactone/nepetalic acid and 1,8-cineole over phenolic constituents in NREO [26,30], which reinforces the need for dedicated DPPH/ABTS/FRAP assays on NREO itself. The available antioxidant data for DMEO and for EOs of other nepetalactone-rich *Nepeta* species are summarized in Table 3.

### 4.2. Antibacterial Activity

DMEO has been reported to exhibit broad-spectrum antibacterial activity against both Gram-positive and Gram-negative bacteria. Disk-diffusion assays produced inhibition zones of 40.0 mm against *Staphylococcus aureus*, *Listeria monocytogenes*, *Escherichia coli*, and *Salmonella* Typhimurium (Table 4), values that exceeded those obtained with the antibiotic combination cefotaxime–clavulanic acid used as a positive control. A smaller inhibition zone (21.0 mm) was recorded against *Pseudomonas aeruginosa*, matching the antibiotic control, while *Bacillus cereus* showed a reduced zone (16.0 mm) relative to the antibiotic (27.0 mm). Minimum inhibitory concentration (MIC) values confirmed this pattern of sensitivity, with DMEO showing the strongest activity against *S. aureus* and *S.* Typhimurium (MIC = 0.78%), followed by *L. monocytogenes* (MIC = 1.56%) and *E. coli* (MIC = 3.125%), whereas *B. cereus* and *P. aeruginosa* remained resistant even at the highest tested concentration (MIC > 100%) [23]. These inhibition zones, obtained with neat DMEO, should nevertheless be interpreted with caution. Inhibition-zone diameters of EOs are strongly influenced by their volatility and limited diffusion in agar, as well as by the EO concentration, solvent, inoculum size, and disk or well conditions, and therefore cannot be directly compared with those of antibiotics tested at standardized concentrations, nor across studies using different protocols. Accordingly, the larger zones recorded for neat DMEO relative to the antibiotic controls reflect the assay conditions rather than a demonstrated superiority over antibiotics, and MIC values provide a more reliable basis for comparison.

An independent study by Ehsani et al. [25] similarly confirmed a stronger effect of DMEO against Gram-positive bacteria, with the lowest MIC and MBC values recorded against *S. aureus* (0.12 and 0.25 mg/mL, respectively); by contrast, *E. coli* and *S.* Typhimurium proved comparatively more resistant, both requiring the same, higher MIC and MBC values of 2.00 and 4.00 mg/mL (Table 5). In the same study, disk-diffusion assays showed dose-dependent increases in inhibition-zone diameter across all four species (8.21–9.53 mm at 10 mg/mL and 9.25–12.15 mm at 15 mg/mL). DMEO produced measurable inhibition zones against *S. aureus*, whereas no measurable zone was obtained with either vancomycin or ampicillin; however, DMEO remained less effective than ampicillin against *E. coli*, *S.* Typhimurium, and *L. monocytogenes*, and less effective than vancomycin against *L. monocytogenes* (Table 4).

Time-kill kinetic analysis further demonstrated that the antibacterial activity of DMEO is both concentration- and exposure-time-dependent. At the MIC, a bactericidal effect was reached within 3 h of contact for *S. aureus* and *S.* Typhimurium, within 4 h for *L. monocytogenes*, and within 12 h for *E. coli*. Increasing the concentration to four times the MIC shortened these intervals substantially, achieving complete bacterial elimination within 1 h for *L. monocytogenes*. These kinetic models, validated with high coefficients of determination (0.93–1), confirm that both the intensity and the speed of the antibacterial effect of DMEO are dose-dependent [23].

Beyond the standard panel of food-borne pathogens, DMEO has also been evaluated against *Enterococcus faecalis*, where it produced only a bacteriostatic effect, inhibiting bacterial growth without exerting bactericidal activity, as reflected in a 12 mm inhibition zone in the disk-diffusion assay, using gentamicin as a positive control [34].

Sheykhjan et al. [22] evaluated the antibacterial potency of *D. moldavica* EO against a broader panel of food-relevant pathogens, comparing the neat oil (DMEO), a 10% dilution (DMEO10%) and a Tween-80 nano-emulsion (DMNEO) against seven bacterial strains using both disk-diffusion and broth-microdilution (MIC/MBC) assays (Table 4 and Table 5). By disk-diffusion, *Bacillus cereus* displayed the highest sensitivity to all three preparations (inhibition-zone diameter of 70.33 ± 0.33 mm for DMEO). As noted above, such exceptionally large zones obtained with the neat oil reflect the assay conditions and should not be interpreted as superior activity relative to the antibiotic controls. By broth microdilution, the lowest MIC and MBC values overall were recorded for *S. aureus* (MIC = 0.312 mg/mL, MBC = 2.5 mg/mL) and *Sh. dysenteriae* (MIC = 0.625 mg/mL, MBC = 5 mg/mL) (Table 5). DMEO consistently outperformed both DMEO10% and DMNEO in absolute terms, reflecting its higher EO concentration; however, the marked reduction in droplet size achieved in the nano-emulsion (136.64 nm) significantly enhanced antibacterial efficacy relative to DMEO10% at the same nominal EO concentration, mirroring the potentiating effect of nanoencapsulation already noted for the antioxidant assays. As in Ehsani et al. [25], susceptibility was consistently higher among Gram-positive strains, a pattern attributed to the absence of an outer lipopolysaccharide membrane, which in Gram-negative bacteria restricts penetration of the hydrophobic EO constituents.

For *N. racemosa* specifically, direct antibacterial data remain scarce. Lungoci et al. [20] incorporated NREO into polyvinyl alcohol–pullulan scaffolds and reported effective antibacterial activity against both *Staphylococcus aureus* and *Escherichia coli*, with treated bacterial cells showing membrane deterioration, increased membrane permeability, cell lysis and agglutination on microscopic examination [20,33]. This appears to be one of the few reports addressing the antibacterial activity of NREO itself, and, as with the antioxidant data above, highlights the absence of dose–response (MIC/MBC) characterization for NREO relative to the more extensively studied DMEO.

Azizian et al. [29] evaluated NREO for antifungal rather than antibacterial activity, reporting that EO concentrations of ≥600 ppm produced a fungicidal effect against *Botrytis cinerea*, *Cladosporium fulvum* and *Pyricularia oryzae*, and that complete inhibition of *Stemphylium botryosum* mycelial growth was achieved at ≥600 ppm specifically with the essential oils of *N. racemosa* and *N. saccharata* [29,31]. While this cannot substitute for antibacterial MIC/MBC data, it indicates that NREO retains measurable antimicrobial activity as a volatile oil, consistent with, and lending some plausibility to, the antibacterial effect reported qualitatively for NREO by Lungoci et al. [20] above.

At the genus level, the antibacterial profile expected for NREO would be consistent with the pattern reported for other nepetalactone-rich *Nepeta* EOs. *N. cataria* EO, for instance, inhibited methicillin-sensitive *S. aureus* at MIC = 0.15–0.22 µL/mL and a cephalosporin-sensitive *E. coli* strain at MIC = 3.17–4.00 µL/mL [16], as shown in Table 6, while the EO of *N. ucrainica* L. ssp. *kopetdaghensis* was markedly more potent against *S. aureus* (MIC = MBC = 14 µg/mL) than against *M. luteus* (MIC = 28 µg/mL, MBC = 55 µg/mL) or *P. aeruginosa* and *E. coli* (MIC, MBC > 150 µg/mL for both) [16]. Likewise, *N. sessilifolia* Bunge EO inhibited *S. aureus*, *K. pneumoniae* and *E. coli* at MIC values between 125 and 500 mg/mL [33,35], and *N. nuda* EO was active against several human- and plant-pathogenic bacteria at MIC values as low as 7.81 mg/mL against the most sensitive strains [33]. These genus-wide trends (pronounced Gram-positive susceptibility and comparatively higher MICs against Gram-negative species such as *E. coli* and *P. aeruginosa*) mirror those obtained for DMEO and, pending direct confirmation, support the plausibility of a similar susceptibility profile for NREO.

### 4.3. EO Mechanisms of Action in DMEO and NREO and Their Main Compounds

The antibacterial efficacy of EOs is attributable to the synergistic interactions among their constituent components, whereby the type and concentration of the chemical entities present determine the specific cellular targets engaged. Bacteria exhibit considerably greater difficulty in acquiring resistance to multicomponent EOs compared with conventional antibiotics, which are typically characterized by a single active chemical entity [36]. EOs demonstrate potent antibacterial activity through the simultaneous targeting of multiple sites within microbial cells, a multitarget mechanism that suppresses bacterial growth without promoting the development of resistance [37]. Their antibacterial effects have been attributed to several interconnected mechanisms, including disruption of the cytoplasmic membrane, alterations in the fatty acid composition of the cell membrane, degradation of cellular structures, and a reduction in the proton motive force accompanied by ATP depletion and the leakage of metabolites and ions across compromised membranes [38].

Apart from the membrane damage documented for NREO incorporated into polymeric scaffolds, the mechanisms discussed below have been established for individual constituents of DMEO and NREO or for EOs of other species, rather than for DMEO and NREO as whole oils. Since synergistic, additive, or antagonistic interactions among EO constituents may substantially modify their biological effects, these mechanisms cannot be directly attributed to the whole oils and should be regarded as indicative until confirmed experimentally.

#### 4.3.1. Direct Antibacterial Mechanisms

The oxygenated monoterpenes that dominate DMEO are biosynthetically interconnected: geranyl acetate is converted to geraniol by geranyl acetate esterase, and geraniol is subsequently oxidized to its aldehyde isomers geranial and neral, which together constitute citral, through the action of geraniol dehydrogenase [39]. Monoterpenes of this class interact with phospholipid membranes, functioning as interstitial impurities within the ordered structure of the lipid bilayer, an interaction closely associated with the hydrophobic character of terpenoid compounds. Andrade-Ochoa et al. [40] reported that Gram-negative bacteria, despite their double-membrane architecture, are consistently more resistant to EOs than Gram-positive species, a finding that challenges a purely hydrophobic model of action, whereas the pronounced hydrophobicity of the mycobacterial cell wall correlates with increased susceptibility. DMEO and NREO are both dominated by monoterpenoid constituents, oxygenated monoterpenes such as geraniol, linalool, geranial, and neral in DMEO, and the iridoid nepetalactone together with 1,8-cineole in NREO, suggesting that their antibacterial potential may fall within this same hydrophobicity-driven framework, although this remains to be confirmed experimentally for the whole oils.

Interference with the cell membrane, comprising disruption of electron transport, ionic gradients, protein translocation, and enzyme-dependent reactions, is considered the predominant mechanism of EO antimicrobial activity [40]. This mechanism has been documented directly for NREO applied as a whole oil, and for linalool, 1,8-cineole, geraniol, and citral as individual constituents, consistently producing membrane depolarization or hyperpolarization, altered permeability and surface charge, and leakage of intracellular nucleic acids and proteins, confirmed ultrastructurally by electron microscopy in several of these studies (Table 7). Among the individual constituents, potency appears to track hydrophobicity rather than functional group alone: the acetate ester geranyl acetate is a more potent antimicrobial than its parent alcohol geraniol, while geraniol itself additionally acts as an efflux-pump inhibitor against multidrug-resistant *Enterobacter aerogenes* [41], illustrating that a single constituent can combine more than one mode of action.

Beyond membrane effects, citral also acts on bacterial genetic material: Gutiérrez-Pacheco et al. [39] reported that citral caused the death of *Escherichia coli* MG1655 primarily through oxidative DNA damage, independent of the tricarboxylic acid cycle, the Fenton reaction, and iron availability (Table 7). This bactericidal DNA-damaging effect is distinct from the antigenotoxic protection essential oils can confer on host cellular DNA, defined as the ability to reduce, counteract, or prevent genetic damage induced by mutagens or genotoxins [42].

Respiratory metabolism, which sustains cellular function through the pentose phosphate pathway, glycolysis, and the tricarboxylic acid cycle, is a second major target: its inhibition impedes bacterial growth and ultimately causes cell death [43]. This has been demonstrated directly for linalool and citral, both of which suppress key metabolic enzymes and reduce intracellular ATP at inhibitory concentrations.

**Table 7:** Mechanisms of action of DMEO, NREO, and their principal constituents.

Category	Compound(s)	Target/Model	Mechanism–Key Finding	Level of Evidence	Reference
**Direct antibacterial mechanisms**	NREO (whole oil)	*Staphylococcus aureus*, *Escherichia coli*	SEM: membrane deterioration, diffusion, lysis, agglutination	Whole EO; in vitro	[20,33]
	Linalool	*Pseudomonas fluorescens* (at MIC)	Membrane: depolarization (68.64% decrease in membrane-potential fluorescence); leakage of alkaline phosphatase, nucleic acids and proteins (3.46-fold increase in nucleic acid absorbance; 81.66% decrease in protein content within 2 h); SEM: deformed, wrinkled, partially lysed cells. Energy metabolism: ATPase activity decreased to 1.0646 U/mg protein (minimum); pyruvate kinase activity decreased by up to 52.57%; succinate/malate dehydrogenase suppressed; respiratory-chain dehydrogenase activity decreased by up to 76.37% at MIC; overall metabolic activity decreased (resazurin assay)	Isolated constituent; in vitro	[44]
	1,8-Cineole	Carbapenemase-producing *Klebsiella pneumoniae*	Increased surface charge and outer-membrane permeability; leakage of nucleic acids/proteins; impaired efflux-pump function (9.4-fold increase in ethidium bromide accumulation); SEM/TEM: corrugated surface, cytoplasmic clearing	Isolated constituent; in vitro	[45]
	Geraniol	– (membrane phospholipid model)	Solvates membrane phospholipids; intracellular leakage; transient loss of membrane organization preceding protein denaturation	Isolated constituent; in vitro	[39]
	Geraniol (as dominant compound, 81.25%, in palmarosa EO)	*Staphylococcus aureus*, MRSA, *Bacillus cereus*, *Enterococcus faecalis* (Gram+); *Pseudomonas aeruginosa* (Gram−)	Agar-diffusion inhibition, supporting geraniol’s membrane mechanism at the whole-oil level	Other species EO; in vitro	[46]
	Geranyl acetate/Geraniol	Multidrug-resistant *Enterobacter aerogenes*	Geranyl acetate is a more potent antimicrobial than geraniol (greater hydrophobicity); geraniol additionally acts as an efflux-pump inhibitor	Isolated constituent; in vitro	[41]
	Citral	*Cronobacter sakazakii* (MIC 0.27–0.54 mg/mL)	Membrane: hyperpolarization (fluorescent probe); SEM: structural membrane damage. Energy metabolism: intracellular ATP decreased from 0.41 to 0.075 µmol/L at MIC (0.054 µmol/L at 2× MIC); intracellular pH decreased; longer lag phase	Isolated constituent; in vitro	[47]
	Citral	*Escherichia coli* MG1655	Oxidative DNA damage, independent of the TCA cycle, the Fenton reaction, and iron availability	Isolated constituent; in vitro	[39]
	Nepetalactone ((Z,E)- and (E,Z)-isomers, as in catnip EO)	5 Gram+ and 9 Gram− species; strongest vs. *Neisseria subflava*, *N. sicca*; moderate vs. *Citrobacter freundii*, *E. coli* O157, *Serratia marcescens*, *S. aureus*	Growth inhibition; both isomers synergistic in the natural EO	Related species EO; in vitro	[48]
**Anti-inflammatory, antioxidant, and anti-virulence mechanisms of major constituents**	Geraniol	Mice, systemic MRSA infection (in vivo)	Decreased bacterial load (blood, lung, liver; dose-dependent); decreased IL-1β, IL-6, and TNF-α; restored SOD and GPx activity; decreased MDA and hydroxyl radical; ameliorated neutrophilic infiltration and alveolar collapse	Isolated constituent; in vivo	[49]
	1,8-Cineole	Inflammatory model	Decreased TNF-α, IL-6, and IL-1β; increased catalase; decreased MDA and ROS; suppressed TLR4/MyD88; decreased phosphorylated IκBα and p65 (NF-κB down regulation)	Isolated constituent; in vitro	[50]
	1,8-Cineole	– (pathway-level mechanism)	Inhibits NF-κB p65 nuclear translocation; activates PPAR-γ signaling; antioxidant activity via Nrf2/Keap1 pathway activation (increased HO-1, NQO1, SOD, catalase, GPx)	Isolated constituent; in vitro and in vivo	[51]
	Geraniol (quorum sensing)	*Pseudomonas aeruginosa* PAO1 (LasR receptor docking)	H-bonds with Thr75/Ser129; competitive LasR inhibition; palmarosa EO: pyocyanin reduced 71%, elastase reduced 51%, biofilm reduced 64%	Isolated constituent; in silico	[46]
	1,8-Cineole (quorum sensing/biofilm)	Several Gram+/Gram− species	Inhibits quorum sensing and biofilm formation; alters cell shape and size	Isolated constituent; in vitro	[51]

Note: MIC = minimum inhibitory concentration; SEM/TEM = scanning/transmission electron microscopy; MRSA = methicillin-resistant Staphylococcus aureus. Whole EO: DMEO or NREO; Related species EO: EO of another *Nepeta* species; Other species EO: EO of an unrelated plant species.

These constituent-level mechanisms are consistent with observations reported for the intact EO of a related species: catnip (*Nepeta cataria*) essential oil, composed predominantly of the (Z,E)- and (E,Z)-nepetalactone isomers, inhibits a broad range of Gram-positive and Gram-negative species, with both isomers acting synergistically [48].

#### 4.3.2. Anti-Inflammatory, Antioxidant, and Anti-Virulence Mechanisms of Major Constituents

Immunomodulatory and antiviral activities have not been evaluated for DMEO or NREO as whole oils in the studies included in this review. The effects discussed in this subsection were documented for individual constituents, mainly in relation to the host inflammatory and oxidative response to bacterial infection and to bacterial virulence, and are presented as mechanistic support for the antibacterial activity rather than as evidence of immunomodulatory activity of the oils themselves.

Beyond their direct bactericidal action, DMEO and NREO constituents also act at the host–pathogen interface, along two mechanistically distinct routes that nonetheless converge in at least one constituent. Rather than killing bacteria outright, they modulate the host’s own inflammatory and oxidative response to infection on one hand, and interfere with the cell-to-cell signaling systems bacteria use to coordinate virulence and biofilm formation on the other. For 1,8-cineole, both routes have been documented from the same body of work: NF-κB signaling is modulated on the host side, dampening the inflammatory response to infection, while a related quorum-sensing regulatory network is suppressed on the bacterial side [51], indicating that a single constituent can act simultaneously on host physiology and on bacterial communication rather than on either alone.

On the host side, geraniol administered to mice with systemic MRSA infection reduced bacterial load in blood, lung, and liver in a dose-dependent manner, lowered pro-inflammatory cytokine release and oxidative damage markers, and restored antioxidant enzyme activity, with concurrent amelioration of neutrophilic infiltration and alveolar collapse in lung tissue [49]. A comparable pattern has been reported for 1,8-cineole in an inflammatory model, where suppression of TLR4 and MyD88 expression reduces NF-κB pathway activation, while the antioxidant response is instead linked to activation of the Nrf2/Keap1 pathway (Table 7); these two upstream pathways together account for the anti-inflammatory and antioxidant effects of 1,8-cineole described more broadly by Hoch et al. [51].

On the bacterial side, geraniol was shown computationally to dock into the LasR quorum-sensing receptor of *Pseudomonas aeruginosa*, forming hydrogen bonds with the Thr75 and Ser129 residues of the binding site and thereby competitively inhibiting the receptor, an effect reflected functionally in reduced production of the virulence factors pyocyanin and elastase and in reduced biofilm formation [46]; 1,8-cineole acts on a related quorum-sensing and biofilm-formation network across several Gram-positive and Gram-negative species, together with alterations in bacterial cell shape and size [51].

## 5. Applications of DMEO and NREO

Although the biological properties of DMEO and NREO have been investigated, data regarding their direct incorporation into diverse product matrices remain limited. A few studies document the use of the plants themselves as ingredients in food products. More broadly, DMEO and NREO have been documented across a wide spectrum of practical applications, spanning food technology, agricultural biocontrol, and cosmetic manufacturing. In addition to their antioxidant and antibacterial activities, DMEO and NREO have been associated with phytotoxic, insecticidal, and sedative properties, which support their potential use in agricultural biocontrol and in pharmaceutical or cosmetic products, as outlined below.

### 5.1. Food Applications

The EOs derived from *Nepeta* species have been widely recognized as promising candidates for food preservation and flavoring applications [52]. Characterized by elevated concentrations of monoterpene hydrocarbons and oxygenated monoterpenes, these EOs have been proposed as food preservatives and antioxidant agents [28].

Investigations into the antimicrobial potential of NREO demonstrated significant inhibitory activity against both Gram-positive and Gram-negative bacterial strains. Biodegradable composite films and hydrogels fabricated from polyvinyl alcohol, pullulan, and NREO exhibited favorable antibacterial properties, with proposed applications in active food packaging for perishable commodities including meat, fish, seafood, fruits, and vegetables; however, these materials were evaluated in vitro, and their application to real food products has not yet been reported [20].

A comparable packaging application has been described for DMEO: blend films based on hydroxypropyl methylcellulose and sodium alginate incorporating DMEO were developed as functional, bio-based alternatives to conventional synthetic packaging materials, with antioxidant activity increasing significantly with essential oil content [53]. Similarly, hydroxypropyl methylcellulose films incorporating sodium alginate and DMEO have been developed as biodegradable active packaging systems, exhibiting enhanced antioxidant activity and improved water barrier properties relative to pure hydroxypropyl methylcellulose films [54]. Overall, the food-related applications of DMEO and NREO documented to date consist mainly of the development and characterization of active packaging materials, whereas their use as preservatives or flavoring agents in real food products remains largely proposed and requires validation in food matrices.

Model studies employing food-origin microorganisms and appropriate sensory evaluations are still required to confirm that *Nepeta* spp. formulations do not adversely affect the organoleptic characteristics of final products [16].

### 5.2. Other Relevant Applications

DMEO has been reported to exhibit strong germination inhibitory activity, indicating potential utility as a naturally derived herbicide. The EO induces excessive hydrogen peroxide accumulation, elevated lipid peroxidation, and electrolyte leakage in weed seedlings, collectively resulting in oxidative stress and growth inhibition, supporting the allelopathic potential of DMEO for bio-pesticide applications [54].

The EOs of the genus *Nepeta* have been identified as effective botanical insecticides, with NREO exhibiting adulticidal and nymphicidal activity against *Bemisia tabaci*. Fumigant bioassay activity against *Sitophilus oryzae* adults has similarly been reported for *N. glomerulosa*, *N. pogonosperma*, and *N. cataria* essential oils. The insecticidal properties of *Nepeta* EOs have been attributed to the presence of 1,8-cineole and nepetalactone, the latter demonstrating repellent, antifeedant, and ovipositional deterrent activity against *Stomoxys calcitrans*. Herbicidal activity has additionally been reported for *Nepeta* EOs, with inhibitory effects on seed germination and shoot growth of both broad-leaf and grassy weed species documented predominantly in Turkey. The application of *Nepeta* EOs as botanical fungicides, insecticides, and herbicides has been highlighted as particularly advantageous given their efficacy against phytopathogens and insect pests; although these EOs have been suggested to have limited adverse effects on non-target organisms, this has not been specifically assessed for NREO and requires experimental confirmation [33].

The EOs of the genus *Nepeta*, particularly those exhibiting antimicrobial and antioxidant activities attributable to compounds such as 1,8-cineole and caryophyllene oxide, have been proposed as promising candidates for novel pharmacological formulations [29,55] and are additionally utilized in the perfume and cosmetic industry, notably in soap manufacturing and as natural flavoring agents [33].

## 6. Toxicological Safety Assessment of DMEO and NREO Major Constituents

To date, comprehensive toxicological studies specifically addressing DMEO and NREO remain scarce in the scientific literature. In the absence of direct toxicological data for these EOs as complex mixtures, the safety assessment of their major constituents is used here as an indicative approach, although toxicological read-across from individual compounds cannot fully predict the toxicity of a chemically complex EO. Given the compositional complexity of both EOs, encompassing 15 to 53 individually resolved compounds by GC–MS analysis, an exhaustive read-across of every detected constituent is neither feasible nor proportionate to its toxicological relevance. The present assessment therefore focuses on the five principal constituents of each EO, selected for their consistently reported dominance in the compositional literature: for DMEO, geranyl acetate, geranial, neral, geraniol, and linalool, which together account for the substantial majority of the oxygenated monoterpene fraction; and for NREO, nepetalic acid, nepetalactone, germacrene D, 1,8-cineole, and palmitic acid.

This approach mirrors the component-based framework used by regulatory bodies such as JECFA and EFSA for the safety evaluation of complex natural flavoring and fragrance mixtures, which combines compositional accounting, structural read-across, and threshold-based approaches rather than exhaustive individual testing of every constituent [56]; minor constituents were therefore not evaluated individually here, although synergistic or additive interactions between them cannot be entirely excluded. It should also be noted that the information summarized below derives largely from regulatory classifications, GHS notifications, and toxicological database entries for the individual constituents, rather than from experimental studies performed on DMEO and NREO; the only experimental data available for the whole oils are the acute toxicity observations reported for DMEO.

Improper use of EOs and plant secondary metabolites may cause adverse effects ranging from mild headache, nausea, or skin and eye irritation to more serious acute oral or inhalation toxicity and, at high doses, hepatic or gastric injury [15], particularly relevant for internal or food-additive use. Specific safety concerns associated with *Nepeta* products have also been highlighted in the literature, underscoring the need for rigorous dose evaluation and regulatory oversight [16,33].

Beyond constituent-level data, whole-EO acute toxicity studies on DMEO have reported a sedative effect in laboratory animals, with reduced motor activity, muscle relaxation, and antinociceptive properties [54]. This is consistent with the sedative pharmacology later documented for linalool, including hypnotic and hypothermic effects linked to cortical glutamate inhibition.

### 6.1. Toxicological Profile of the DMEO Major Constituents

Geranyl acetate, a major constituent of DMEO, has a low acute toxicity profile, with oral LD50 values of 6330 mg/kg in rats and an LDLo of 8000 mg/kg in mice. Human patch testing showed only mild skin irritation, though the compound is classified under GHS as a skin irritant (H315) and sensitizer (H317), and is listed as a fragrance allergen under Annex III of the EU Cosmetics Regulation (EC) No. 1223/2009 [57]. It is recognized as safe for food use, with a Joint FAO/WHO Expert Committee on Food Additives (JECFA) Acceptable Daily Intake (ADI) of 0–0.5 mg/kg body weight and Generally Recognized As Safe (GRAS) status under United States Food and Drug Administration (FDA) regulation (21 CFR 182.60) [58]. Chronic National Toxicology Program (NTP) carcinogenicity studies in rats and mice found no evidence of carcinogenic activity, with only marginal, non-significant findings at low doses. Genotoxicity results were mixed: the Ames assay was negative, but chromosomal aberrations and mutagenic activity were reported in other in vivo assays, so the overall genotoxic potential cannot be considered uniformly negative [58].

Geranial, the (E)-isomer of citral, is assessed jointly with neral under the designation “citral,” since the two isomers co-occur naturally and share a similar toxicological profile. Acute toxicity is low, with oral LD50 values generally exceeding 1000 mg/kg body weight in rodents (range approximately 1440–6000 mg/kg). Repeated-dose and reproductive/developmental studies in rats established no-observed-adverse-effect levels (NOAELs) of 200–570 mg/kg/day, and an inhalation teratogenicity study found no effect up to 423 mg/m^3^. Chronic NTP feeding studies found no carcinogenic activity in rats or male mice, though equivocal evidence (increased lymphoma) was reported in female mice at the highest dose; citral is not listed as a carcinogen by IARC. Genotoxicity testing was largely negative across Ames, chromosomal aberration, and micronucleus assays. Citral is a recognized skin irritant (H315) and sensitizer (H317), with eye irritation (H319) reported in about 85% of notifications, and is listed as a contact allergen under EU Cosmetics Regulation (EC) No. 1223/2009 [57]. Regulatory ADIs vary between assessments (EFSA: 0.05 mg/kg bw/day; JECFA-derived: 0–0.5 mg/kg bw/day), and citral is GRAS for flavoring use under US FDA regulation (21 CFR 182.60) [59].

Neral, the (Z)-isomer of citral and a major constituent of DMEO, shows low acute toxicity, with oral LD50 values of approximately 4895–6000 mg/kg in rodents and dermal LD50 values of 2000–2250 mg/kg; intraperitoneal LD50 was markedly lower (147–215 mg/kg), reflecting strong route-dependence. Subchronic and reproductive/developmental studies established NOAELs of 60–200 mg/kg/day, and an inhalation NOAEC of 211.7 mg/m^3^ was reported. Genotoxicity evaluation of neral was negative, and neral is not listed as a carcinogen by IARC. It is classified under GHS as a skin irritant (H315) and sensitizer (H317), with eye irritation (H319) reported in about 63% of notifications. The available evidence supports its safe use as a flavoring substance at current exposure levels [60].

Geraniol, one of the major constituents of DMEO, has low acute toxicity (oral LD50 3600 mg/kg in rats) but marked species-dependent differences in irritation and sensitization: it was non-irritating and non-sensitizing in standard animal models, yet human patch testing showed severe irritation at 32% concentration and documented cases of allergic contact dermatitis. Sensitization rates rise from 0.13% for pure geraniol to 0.55% for its autoxidized form, given that oxidation converts geraniol into the more sensitizing geranial and neral; it is listed as a fragrance allergen under EU Cosmetics Regulation (EC) No. 1223/2009. A rare fatal case of citronella oil ingestion (93% geraniol) is documented in the literature. Subchronic dietary studies in rats found no adverse effects up to 500 mg/kg/day. Genotoxicity data are inconsistent across EFSA assessment years, though a 2022 RIFM assessment concluded geraniol is not genotoxic, with a Margin of Exposure above 100 and no expected phototoxic potential [61]. Geraniol is not listed as a carcinogen by IARC. Under GHS, it carries the signal word “Danger” as a skin irritant (H315), sensitizer (H317), and serious eye damage hazard (H318, more severe than the other DMEO constituents), and is GRAS for flavoring use under US FDA regulation (21 CFR 182.60) [62].

Linalool, one of the five principal constituents of DMEO, has low acute toxicity via oral and dermal routes (oral LD50 2790 mg/kg in rats; dermal LD50 5610 mg/kg) but greater sensitivity via injection (intraperitoneal LD50 307–340 mg/kg). It was irritating to rabbit and guinea pig skin but not a sensitizer in guinea pig maximization tests; in humans, sensitization rates were low (0.17–0.8%, rising to nearly 10% in pre-sensitized individuals), among the lowest of 26 fragrance allergens tested. It nonetheless remains a listed contact allergen, given that a fraction oxidizes on air/skin exposure to more potent sensitizing hydroperoxides. A 28-day oral study in rats established a NOAEL of 160 mg/kg/day for liver and kidney effects. Genotoxicity testing across a full battery, including EFSA’s 2020 evaluation, was consistently negative, and no carcinogenicity classification is listed. Linalool exerts marked, dose-dependent sedative effects in rodents (hypnotic, anticonvulsant, hypothermic, via cortical glutamate inhibition), while causing toxicosis in cats. It is registered by the US EPA as a biopesticide, practically non-toxic to birds and fish. Under GHS, linalool carries the signal word “Warning” as a skin irritant (H315), sensitizer (H317, notably lower than geraniol or citral), and eye irritant (H319), and is GRAS for flavoring use under US FDA regulation (21 CFR 182.60) [63].

### 6.2. Toxicological Profile of the NREO Major Constituents

Nepetalic acid, reported as the predominant constituent (up to 55.5%) of NREO in one compositional analysis and structurally related to nepetalactone as a biosynthetic precursor, currently lacks any dedicated toxicological characterization. No acute toxicity, GHS classification, carcinogenicity, or genotoxicity data could be identified in PubChem or associated regulatory databases (ECHA, EFSA), representing a notable data gap given its high abundance in some NREO chemotypes. Available information is limited to biochemical characterization: nepetalic acid is documented as a human metabolite and as a naturally occurring constituent of black tea (*Camellia sinensis*). This circumstantial evidence of historical exposure does not substitute for formal toxicological testing and underscores the need for targeted investigation of this constituent rather than reliance on read-across from the structurally related but pharmacologically distinct nepetalactone [64].

Nepetalactone, the principal bioactive constituent of NREO, demonstrates a favorable toxicological profile based on the available data. The only ECHA GHS notification indicates it does not meet hazard classification criteria, though this is based on a single industry report of limited evidentiary weight. The only acute toxicity data derive from an intraperitoneal study in mice, reporting an LD50 of 1550 mg/kg body weight, with altered sleep time, somnolence, and ataxia; this route does not reflect realistic human exposure, and the value suggests relatively low acute toxicity [65]. No dedicated in vivo carcinogenicity or chronic toxicity data were identified. A 2026 toxicokinetic study combining in silico ADMET modeling with in vitro rat and human liver microsome assays partially addresses this gap, reporting rapid hepatic clearance (over 85% depletion within 120 min via both oxidative and glucuronidation pathways) with comparable kinetics across species, no predicted genotoxic or mutagenic concern, and moderate hepatotoxic potential for the parent compound and its Phase I metabolites; hepatotoxic potential was substantially reduced for the Phase II glucuronide/sulfate conjugates [66].

Germacrene D, a sesquiterpene hydrocarbon among the dominant constituents of NREO (up to 7.4%), has been evaluated by EFSA within Flavoring Group Evaluation 25 across three assessments (2008, 2010, 2011), with genotoxicity recorded as “not determined” in all three, indicating the endpoint remains uninvestigated rather than negative. ECHA hazard notifications are inconsistent: about 60% classify it under Acute Toxicity Category 4 (H302) and Aspiration Hazard Category 1 (H304), while about 40% report that it does not meet GHS criteria at all, likely reflecting variable purity of tested batches. No quantitative LD50 or carcinogenicity data were identified for this compound [67].

1,8-cineole, a prominent constituent of NREO, has an extensively characterized toxicological profile. Clinical reports in children found ingestion of 2–3 mL caused mild sedation, while over 5 mL was associated with serious CNS depression or coma. Acute oral LD50 values in rats ranged from 2480 to 4700 mg/kg, and the dermal LD50 exceeded 2000 mg/kg. Subchronic gavage studies established a NOAEL of 600 mg/kg bw/day, with reversible liver changes at higher doses and a male-rat-specific renal finding considered not relevant to humans. Reproductive/developmental and long-term carcinogenicity studies found no adverse effects, and EFSA genotoxicity evaluations were negative (2008, 2011). Local irritation testing found 1,8-cineole to be non-irritant to skin and eyes. Under GHS, it is classified as a flammable liquid (H226) and, notably, a skin sensitizer (H317, ~84% of notifications). 1,8-cineole is recognized as safe for food use by JECFA and permitted as a food additive under US FDA regulation (21 CFR 172.515) [68].

Palmitic acid (n-hexadecanoic acid), the major saturated fatty acid in NREO, has a comparatively benign toxicological profile, reflecting its ubiquity in plant and animal lipids. The CIR Expert Panel considers it safe for cosmetic use, subject to concentration qualifications. Acute toxicity is low by conventional routes, with only transient CNS and gastrointestinal effects in rats at high oral doses; an intravenous LD50 of 57 mg/kg in mice reflects the route-specific sensitivity of systemic administration rather than realistic exposure. Dermal and ocular irritation in rabbits and humans was mild and concentration-dependent. Chronic high-dose feeding can contribute to hyperlipemia and atherosclerotic changes, and palmitic acid is mechanistically implicated in lipotoxicity pathways when present in excess, though it also fulfills essential physiological functions, including lung surfactant biosynthesis in infants. IARC found no indication of carcinogenic activity, and an older, methodologically inadequate mouse study reporting tumor-promoting activity is not considered reliable. Most industry GHS notifications (66.5%) report that palmitic acid does not meet hazard classification criteria; where assigned, hazard statements are limited to mild, reversible endpoints. Palmitic acid is listed by the US EPA Safer Choice program in its lowest-concern category and is a permitted food additive under US FDA regulation (21 CFR 172.860) [69].

Several additional factors should be considered when extrapolating these constituent-level data to the whole oils. First, mixture effects may arise from additive or synergistic interactions among constituents; for example, geraniol, citral, geranyl acetate, and linalool are all skin sensitizers, and their combined presence in DMEO may result in a higher sensitization potential than that of any single constituent, a concern further increased by the formation of more sensitizing oxidation products during storage. Second, minor constituents, which were not assessed individually, may also contribute to the overall toxicity of the oils. Third, most of the data summarized above derive from acute or short-term studies, whereas the effects of repeated or long-term exposure to the whole oils remain uninvestigated. Finally, the relevance of each endpoint depends on the route of exposure: skin irritation and sensitization are most relevant to dermal and cosmetic uses, eye irritation and aspiration hazards to handling and inhalation, and systemic toxicity to oral or food-related uses, for which the regulatory acceptance of the individual constituents as flavorings does not automatically extend to the whole oils.

Taken together, the read-across evaluation of the ten principal constituents identified in DMEO and NREO, summarized comparatively in Table 8, indicates that both essential oils are composed predominantly of compounds with low acute toxicity, an absence of confirmed genotoxic or carcinogenic potential, and long-standing regulatory acceptance for food, flavoring, and cosmetic use. The main safety concern relates not to systemic or carcinogenic toxicity, but to dermal and ocular irritation and skin sensitization, most consistently documented for geraniol, citral, and, to a lesser extent, geranyl acetate and linalool, several of which are recognized fragrance allergens under EU Cosmetics Regulation (EC) No. 1223/2009. This is directly relevant to dermal or cosmetic applications of DMEO, and less relevant to inhalation or flavoring use. Two limitations should be acknowledged: nepetalic acid, despite being the predominant constituent in some NREO chemotypes, currently lacks any dedicated toxicological data, and the profile of an essential oil as a whole mixture cannot be fully predicted from its individual constituents alone, given possible synergistic or antagonistic interactions. The available constituent-level evidence therefore suggests, but does not establish, a favorable safety profile for DMEO and NREO at exposure levels consistent with their traditional uses; for NREO in particular, this conclusion remains tentative given the absence of data for nepetalic acid and the limited data for nepetalactone and germacrene D, underscoring the need for dedicated studies on the oils themselves.

## 7. Essential Oils Against *Helicobacter pylori*

### 7.1. Helicobacter pylori: Pathogenesis and Rationale for Alternative Therapies

*Helicobacter pylori*, a Gram-negative, microaerophilic, spiral-shaped bacterium, colonizes the gastric mucosa of a substantial proportion of the global population and, once acquired in childhood, persists for life if untreated [70]. Colonization induces chronic-active gastritis in essentially all infected hosts and constitutes the principal etiological factor for peptic ulcer disease, gastric adenocarcinoma, and gastric MALT lymphoma; the World Health Organization classified *H. pylori* as a Group I carcinogen in 1994, and approximately 90% of gastric cancer cases are attributable to the infection [70,71]. Pathogenesis relies on a suite of virulence factors, urease-mediated neutralization of gastric acidity, flagella-driven motility, adhesion via outer membrane adhesins, and the *cag* pathogenicity island/CagA and VacA toxins that trigger inflammatory and oncogenic signaling [70,71].

Standard eradication therapy combines a proton pump inhibitor with two or more antibiotics, most commonly clarithromycin, amoxicillin, and metronidazole. However, clarithromycin resistance now reaches 15–30% worldwide, metronidazole resistance exceeds 25% in most regions, and levofloxacin resistance has reached approximately 20% in Europe, collectively driving eradication failure to clinically unacceptable levels, with adverse events in 30–70% of treated patients and recurrence rates exceeding 50% in some settings due to antibiotic-induced gut dysbiosis [70,72]. These limitations have generated substantial interest in plant-derived essential oils as alternative or adjuvant antimicrobial agents [73]; notably, *Cymbopogon citratus* (lemongrass) essential oil did not elicit resistance in *H. pylori* even after ten sequential passages, in marked contrast to the rapid resistance development observed with clarithromycin under identical conditions [74].

### 7.2. Selectivity of Essential Oils Toward H. pylori

A critical consideration in the therapeutic application of EOs against *H. pylori* is their selectivity: the capacity to inhibit the pathogen without harming beneficial gut microbiota, particularly lactic acid-producing *Lactobacillus* spp. [72]; traditional-style aqueous plant-extract preparations have been shown to preserve *Lactobacillus* viability while retaining, or even potentiating, anti-*H. pylori* activity, in contrast to organic extracts [72]. At the level of EO composition itself, selectivity is strongly chemotype-dependent: Piasecki et al. [74] showed that among *Mentha* EOs, the menthol-rich chemotype matched the reference antibiotic metronidazole in activity against clinical strains, followed by the carvone-rich chemotype, whereas 1,8-cineole, despite being present in all nine oils examined, showed essentially no activity against clinical isolates, demonstrating that specific chemical composition, rather than botanical origin or compound ubiquity, determines anti-*H. pylori* potential. Probiotic *Lactobacillus* strains naturally present in the stomach may independently inhibit *H. pylori* urease activity and improve eradication rates when added to standard therapy [75].

Beyond therapeutic selectivity, incorporating EOs into food matrices as functional antimicrobial agents is constrained by their poor water solubility and high volatility [73], though bactericidal activity of several EO constituents increases significantly at pH 4.0 relative to neutral pH, a finding directly relevant to acidic, fermented, and functional food and beverage matrices approximating gastric conditions. Formulation can meaningfully offset these constraints: a cinnamon EO nano-emulsion showed substantially improved activity against *H. pylori* (MBC 60–470 µg/mL) compared with the oil alone (MBC 140–2340 µg/mL), demonstrating that food-compatible nano-emulsion formulation can enhance efficacy in aqueous food systems [73].

### 7.3. Synergistic Effects of EOs in Anti-H. pylori Activity

EO activity against *H. pylori* frequently exceeds what is predicted from individual constituents alone, reflecting synergistic interactions with practical relevance: effective inhibition at lower constituent concentrations improves the sensory acceptability of EO-containing products. Korona-Głowniak et al. [76] showed, through principal component analysis of 26 EOs, that the most active group, lemongrass, lemon balm, and cedarwood, derived its potency from distinct chemical classes (monoterpene alcohols/aldehydes and sesquiterpenoids, respectively), indicating that synergism among heterogeneous constituents underlies observed activity; binary and ternary EO mixtures produced 2- to 4-fold MIC reductions compared with single oils, and this synergism extends in vivo: a 2:1 mixture of *Satureja hortensis* and *Origanum vulgare* subsp. *hirtum* EOs achieved substantial eradication in a mouse model, with only 30% of treated animals remaining infected [73]. Piasecki et al. [74] further demonstrated synergistic-to-additive interactions between the most active mint EOs and metronidazole (FICI 0.28–0.625) against clinical strains, with their major constituents, menthol and carvone, producing even stronger synergy (FICI 0.094–0.31), while combinations with clarithromycin were only additive (FICI 0.51–1.00); rosmarinic acid, one of the most active anti-*H. pylori* phytochemicals characterized to date, has likewise been shown to potentiate antibiotic activity against *H. pylori* [77]. Collectively, these findings support EO–antibiotic and EO–EO combinations as a rational strategy for enhancing efficacy while reducing the concentrations of organoleptically impactful constituents.

Realizing these synergistic gains in practice, however, is constrained by the poor aqueous solubility, high volatility, and limited gastric bioavailability of EO constituents, which explain why in vivo efficacy often falls short of in vitro potency [73]. Some constituents nonetheless show favorable gastric behavior: the phenylpropanoids cinnamaldehyde and eugenol exhibit bactericidal activity against *H. pylori* that increases under acidic conditions (pH 4.0) relative to neutral pH, while patchouli alcohol retains bactericidal activity across a range of physiological pH values. Even so, oral administration of EOs to *H. pylori*-infected mice did not consistently reduce bacterial density across several studies, with complete eradication achieved in only a minority of animals [73], underscoring that synergistic in vitro potency does not automatically translate into in vivo efficacy without adequate delivery.

Encapsulation, particularly nano-emulsion systems, substantially improves stability and activity under these conditions, as shown for both cinnamon EO and the antibiotic erythromycin against *H. pylori*, while mucus-penetrating nanoparticle carriers offer a further route to the firmly adherent mucus layer where the bacterium resides [70,75]. Co-formulation strategies can also amplify potency without dedicated nanocarrier technology: combining *Thymus capitatus* EO with monolaurin, a lipid-derived agent, yielded substantially higher activity (MIC 31 µg/mL) than the EO alone (500 µg/mL), a further illustration of how combining constituents, whether EO–EO, EO–antibiotic, or EO–lipid, consistently outperforms single agents [73].

EOs are generally recognized as safe at typical use levels, with irritation of mucous membranes, eyes, and skin the principal concern, particularly for phenolic and aldehyde-rich constituents [71]. A *Salvia officinalis* EO demonstrated only moderate anti-*H. pylori* activity, with the authors recommending further in vivo investigation before any safety conclusions can be drawn [77], and complementary brine shrimp lethality screening indicated that aqueous preparations are consistently less toxic than organic extracts, reinforcing that extraction and formulation choices affect host safety as well as antimicrobial selectivity [72]; appropriate dosing, formulation, and route of administration remain central to balancing safety and efficacy at anti-*H. pylori* concentrations [74].

### 7.4. A Research Perspective: DMEO and NREO Against H. pylori

Chemotype-dependent selectivity, together with the structure–activity relationships emerging across the EOs reviewed above, offers a rational basis for anticipating anti-*H. pylori* potential in EOs not yet directly evaluated against this pathogen. Two EOs of current interest, DMEO and NREO, merit consideration in this light, given the correspondence between their characterized chemical composition and constituents already associated with anti-*H. pylori* activity in the literature reviewed above.

DMEO is characterized by a profile dominated by the monoterpene aldehydes geranial and neral, alongside the monoterpene alcohols geraniol and linalool and, in most chemotypes, geranyl acetate as the single most abundant constituent. Citral has been repeatedly identified among the most potent anti-*H. pylori* constituents characterized to date, alongside carvacrol, isoeugenol, nerol, and sabinene [71], with a reported MBC of 40 µg/mL [73]; EOs in which citral’s constituent aldehydes predominate, such as lemongrass (*Cymbopogon citratus*), have likewise ranked among the most active EOs against the reference strain in large comparative screens, although their activity against clinical isolates was comparatively more moderate [76]. Geraniol, also a major DMEO constituent, has independently demonstrated anti-*H. pylori* activity (MBC 100 µg/mL) and a significant anti-ulcer effect together with a 50% reduction in infection in vivo when administered to rats alone or with clarithromycin [73]. El-Sherbiny and Elbestawy [71] further proposed that monoterpenes combining both alcohol and aldehyde functional groups, exemplified precisely by lemongrass and lemon EOs, display the highest anti-*H. pylori* activity among surveyed constituents. Given that DMEO’s dominant constituents span exactly these two functional classes, it could be hypothesized that DMEO represents a chemically rational candidate for anti-*H. pylori* application; this remains to be substantiated through direct testing of the oil itself, since the cited evidence pertains to isolated constituents and to compositionally analogous, but botanically distinct EOs.

NREO is typically dominated by nepetalactone stereoisomers, with 1,8-cineole and germacrene D as recurrent but compositionally variable secondary constituents. A direct precedent exists at the genus level: *Nepeta argolica* subsp. *dirphya* EO, dominated by nepetalactone isomers, together with *N. camphorata* oil, inhibited *H. pylori* growth (MIC 128 µg/mL) in the only study to date assessing *Nepeta* oils directly against this pathogen [31,73]; isolated nepetalactone stereoisomers themselves showed MIC/MBC values of 64 and 128 µg/mL [73]. Independently, nepetalactone isomers from other *Nepeta* species have shown antibacterial and antibiofilm activity against Gram-negative bacteria, including a resistant strain of *Pseudomonas aeruginosa*, with stereochemistry differentiating potency, mirroring the isomer-dependent activity reported against *H. pylori* itself [78]. This constitutes a research perspective, grounded in genus-level precedent and constituent-level data rather than direct testing of *N. racemosa*, suggesting the nepetalactone-dominant fraction of NREO may warrant investigation.

Nevertheless, 1,8-cineole, frequently a substantial NREO component (up to 70% in some accessions), shows limited anti-*H. pylori* relevance: Piasecki et al. [74] found it essentially inactive against clinical isolates despite its presence in all nine mint oils tested, and a *Salvia officinalis* oil dominated by 1,8-cineole displayed only moderate activity [77]. The nepetalactone fraction therefore appears more relevant to NREO’s anti-*H. pylori* potential than its 1,8-cineole content. The available evidence, grouped according to its relevance to DMEO and NREO, is summarized in Table 9.

Accordingly, future studies should prioritize the direct evaluation of chemically characterized DMEO and representative NREO chemotypes, including (i) determination of MIC and MBC values against both reference and clinical *H. pylori* strains, including antibiotic-resistant isolates; (ii) assessment of urease inhibition and antibiofilm activity; (iii) evaluation of cytotoxicity toward gastric epithelial cells; (iv) investigation of synergy with standard antibiotics such as clarithromycin, amoxicillin, and metronidazole; and (v) subsequent in vivo validation in appropriate infection models.

## 8. Limitations and Future Perspectives

The present review has several methodological limitations. As a narrative review, it did not include a formal assessment of the methodological quality of the included studies, and the search was restricted to English-language publications and to four databases, so that relevant studies published in other languages or indexed elsewhere may have been missed. In addition, publication bias cannot be excluded, since studies reporting positive biological activities are more likely to be published than those reporting negative or inconclusive results. As the number of primary studies on these EOs increases, future reviews could benefit from a systematic protocol including formal quality assessment and multilingual searches.

Substantial heterogeneity in plant origin, chemotype, extraction and analytical methods, and experimental protocols for assessing biological activity limits direct comparison of reported results. Standardized protocols for EO extraction, chemical characterization, and biological testing, including the use of reference strains, recognized standards, and MIC/MBC determination, would allow more reliable comparisons across studies.

The evidence is also considerably stronger for *D. moldavica* than for *N. racemosa*, for which direct antibacterial and toxicological data remain scarce, and findings from other *Nepeta* species cannot be directly extrapolated to *N. racemosa*. Dedicated studies on chemically characterized NREO samples, with explicit reporting of their chemotype, are therefore needed to establish its biological activities directly.

In addition, most toxicological information concerns individual constituents rather than the complete essential oils. Toxicological studies on the whole oils, including cytotoxicity, gastrointestinal safety, repeated-dose exposure, and sensitization potential, as well as targeted investigation of nepetalic acid, are required.

The food-related applications documented to date also consist mainly of the development of active packaging materials, while the use of these EOs in real food products remains largely unexplored. From a food application perspective, particular attention should be given to effective concentrations, sensory acceptability, stability, interactions with food matrices, and appropriate delivery systems.

Most importantly, direct evidence for anti-*H. pylori* activity of DMEO and NREO is currently insufficient; therefore, their potential in this context should be considered hypothesis-generating and requires direct experimental validation. Direct testing of chemically characterized DMEO and representative NREO chemotypes against reference and clinical *H. pylori* strains, including antibiotic-resistant isolates, together with the investigation of their interactions with conventional antibiotics and subsequent in vivo validation, represents the main research priority.

## 9. Conclusions

This review highlights *Dracocephalum moldavica* and *Nepeta racemosa* essential oils as promising, yet unequally characterized, sources of bioactive compounds with potential relevance to food and health-related applications. DMEO has a comparatively stronger evidence base for antioxidant and antibacterial activities, whereas conclusions regarding NREO remain constrained by the limited number of direct biological studies and by its marked chemotypic variability.

From a food perspective, their volatile composition and antimicrobial and antioxidant properties support further investigation of these EOs as potential natural ingredients for food preservation or functional formulations, while their relevance to *Helicobacter pylori* remains a hypothesis-generating research perspective that requires direct experimental validation. Addressing the gaps identified in this review would clarify whether DMEO and NREO can progress from promising phytochemical sources to safe and effective ingredients with potential applications at the interface of food preservation, functional foods, and complementary strategies targeting *H. pylori*.

## Figures and Tables

**Table 1:** Comparative chemical composition of DMEO across different studies.

Chemical Class	Compounds (%)	Reference
**Monoterpene hydrocarbons**	Limonene: 0.11%; 1,3,6-Octatriene: 0.05–0.15%	[22]
	p-Cymene: 2.7%; γ-Terpinene: 1.1%; Myrcene: 0.3%; (E)-β-Ocimene: 0.1%; Sabinene: tr%; Limonene: tr%; (Z)-β-Ocimene: tr%	[11]
	α-Terpinene: 3.12%; Limonene: 1.35%; p-Cymene: 0.92%; β-Pinene: 0.86%; Sabinene: 0.42%; γ-Terpinene: 0.17%; Myrcene: 0.04%	[24]
	Myrcene: 0.10%	[25]
**Oxygenated monoterpenes**	Geranyl acetate: 53.2%; Geranial: 16.8%; Neral: 10.7%; Linalyl acetate: 7.9%; Neryl acetate: 4.6%; Linalool: 1.6%; Borneol: 0.6%; trans-Isocitral: 0.4%; 6-Methyl-5-hepten-2-one: 0.1%; Nerol oxide: 0.1%; Terpinen-4-ol: 0.1%; Lavandulyl acetate: 0.1%; Methyl geranate: 0.1%	[23]
	Geranyl acetate: 32.57%; Geranial: 24.50%; Neral: 22.65%; Geraniol: 8.78%; Neryl acetate: 3.44%; Nerol: 2.36%; 6-Methyl-5-hepten-2-one: 0.95%; Linalool: 0.82%	[21]
	Geranial: 33.94%; Neral: 22.14%; Geranyl acetate: 19.27%; Neryl acetate: 2.19%; Linalool: 1.76%; Rose furan oxide: 1.31%; Geraniol: 1.29%; 6-Methyl-5-hepten-2-one: 1.01%; 2,6-Octadienoic acid: 0.42%; Nerol oxide: 0.37%; Linalool oxide: 0.22%	[22]
	Geranyl acetate: 44.9%; Geraniol: 20.2%; Geranial: 9.3%; Neral: 7.6%; Linalool: 4.1%; Neryl acetate: 2.6%; Nerol oxide: 1.3%; 6-Methyl-5-hepten-2-one: 1.2%; cis-Linalool oxide: 0.6%; Isogeranial: 0.4%; α-Terpineol: 0.3%; Thymol: 0.3%; trans-Linalool oxide: 0.1%; (E)-β-Damascenone: 0.1%; Methyl geranate: 0.1%; Piperitone: tr%; (E)-β-Ionone: tr%	[11]
	Geranyl acetate: 36.62%; Geraniol: 24.31%; Neral: 16.25%; Geranial: 11.21%; Thymol: 1.41%; Carvone: 1.14%; Neryl acetate: 0.91%; Linalool: 0.81%; Linalool oxide: 0.64%; cis-Chrysanthenol: 0.48%; α-Terpineol: 0.36%; Nerol: 0.35%	[24]
	Geranial: 28.52%; Neral: 21.21%; Geraniol: 19.60%; Geranyl acetate: 16.72%; Nerol: 1.86%; Neryl acetate: 1.76%; Linalool: 0.82%; 6-Methyl-5-hepten-2-one: 0.77%; Methyl geranate: 0.17%; Piperitone: 0.14%; Ethyl nerolate: 0.11%; Linalool oxide: 0.08%; Bergamal: 0.07%	[25]
**Sesquiterpene hydrocarbons**	(E)-Caryophyllene: 0.6%; γ-Muurolene: 0.4%; α-Copaene: 0.2%; α-Humulene: 0.1%; (E)-β-Farnesene: 0.1%; (E,E)-α-Farnesene: 0.1%	[23]
	Germacrene D: 0.41%; (E)-Caryophyllene: 0.35%; (E,E)-α-Farnesene: 0.16%; δ-Cadinene: 0.16%; Bicyclogermacrene: 0.14%	[21]
	(E)-Caryophyllene: 0.88%; α-Copaene: 0.51%; (E,E)-α-Farnesene: 0.30%; α-Humulene: 0.28%; δ-Cadinene: 0.21%; Germacrene D: 0.14%; Bicyclogermacrene: 0.10%; α-Calacorene: 0.10%	[22]
	β-Bourbonene: 0.1%; cis-Muurola-4(14),5-diene: 0.1%	[11]
	(E)-Caryophyllene: 0.51%; Germacrene D: 0.47%; β-Elemene: 0.15%; α-Copaene: 0.12%	[24]
	Germacrene D: 0.25%; (E)-Caryophyllene: 0.13%; α-Copaene: 0.10%; δ-Cadinene: 0.06%	[25]
**Oxygenated sesquiterpenes**	Caryophyllene oxide: 0.3%	[23]
	Spathulenol: 0.20%	[21]
	Caryophyllene oxide: 0.43%	[22]
	Caryophyllene oxide: 0.17%; Viridiflorol: 0.02%	[24]
	Caryophyllene oxide: 0.24%; Spathulenol: 0.10%	[25]
**Others (non-terpenoids)**	1-Octen-3-ol: 0.51%	[21]
	Hexadecanoic acid: 0.83%; 1-Octen-3-ol: 0.22%; 2-Pentadecanone: 0.16%; Phthalic acid: 0.12%; Benzene acetaldehyde: 0.10%	[22]
	Benzene acetaldehyde: 0.5%; 1-Octen-3-ol: 0.1%	[11]
	1-Octen-3-ol: 0.25%; Benzene acetaldehyde: 0.20%	[25]

Note: Compounds within each source are listed in decreasing order of relative abundance (%); tr = trace amount (<0.1%).

**Table 2:** Comparative chemical composition of NREO across different studies.

Chemical Class	Compounds (%)	Reference
**Monoterpene hydrocarbons**	β-Pinene: 0.19%	[26]
	β-Pinene: 1.2%; γ-Terpinene: 0.4%; α-Pinene: 0.3%	[27]
	(Z)-β-Ocimene: 10.4%; α-Pinene: 10.2%; β-Pinene: 3.1%; Camphene: 2%; α-Phellandrene: 1.1%; p-Cymene: 0.5%; α-Terpinene: 0.4%; Limonene: 0.2%; α-Thujene: 0.1%	[28]
	Limonene: 0.8%; β-Pinene: 0.3%; (Z)-β-Ocimene: 0.2%; α-Pinene: 0.1%; Sabinene: 0.1%; p-Cymene: 0.1%; (E)-β-Ocimene: 0.1%	[29]
	(Z)-β-Ocimene: 1.1%; β-Pinene: 0.9%; α-Pinene: 0.4%; β-Myrcene: 0.2%	[20]
	(Z)-β-Ocimene: 1.37%; β-Pinene: 0.77%; β-Myrcene: 0.37%; α-Pinene: 0.27%	[30]
	(Z)-β-Ocimene: 9.5%; β-Pinene: 1.1%; α-Pinene: 0.4%; δ-2-Carene: 0.3%	[16] Sample A
	(Z)-β-Ocimene: 1.1%; β-Pinene: 1.1%; α-Pinene: 0.4%; Sabinene: 0.3%	[16] Sample B
**Oxygenated monoterpenes**	4aα,7α,7aα-Nepetalactone: 31.7%; Linalool: 0.39%; trans-Pinocarveol: 0.33%; α-Terpineol: 0.24%; Myrtenal: 0.16%; Myrtenol: 0.14%; Terpinen-4-ol: 0.14%;; Pinocarvone: 0.1%	[26]
	1,8-Cineole: 70.9%; 4aα,7α,7aα-Nepetalactone: 5.3%; Citronellol: 4.3%; 4aα,7α,7aβ-Nepetalactone: 3%; α-Terpineol: 1.7%; 4aβ,7α,7aβ-Nepetalactone: 1.5%; γ-Terpineol: 1.4%; trans-Pinocarveol: 1%; Terpinen-4-ol: 1%; Linalool: 0.5%; α-Campholenal: 0.3%; Pinocarvone: 0.3%; Cumin aldehyde: 0.3%; cis-p-Menth-2-en-1-ol: 0.2%; trans-Verbenol: 0.2%	[27]
	4aα,7α,7aα-Nepetalactone: 28.1%; 4aβ,7α,7aβ-Nepetalactone: 20.5%; 4aα,7α,7aβ-Nepetalactone: 7.4%; α-Terpineol: 2%; Piperitone: 1.8%; Bornyl acetate: 0.3%	[28]
	4aα,7α,7aβ-Nepetalactone: 91%; 4aβ,7α,7aβ-Nepetalactone: 1.5%; 1,8-Cineole: 0.3%; 4aα,7α,7aα-Nepetalactone: 0.1%	[29]
	Nepetalic acid: 55.5%; 1,8-Cineole: 10.7%; 3-Hydroxy-(4S,4aS,7S,7aS)-Dihydronepetalactone: 5.4%; (4aS,7S,7aS)-trans,cis-Nepetalactone: 5%; (4aS,7S,7aR)-cis,trans-Nepetalactone: 1%; Linalool: 0.9%; Terpinen-4-ol: 0.4%; Linalyl acetate: 0.3%	[20]
	Nepetalic acid: 54.33%; 1,8-Cineole: 9.87%; 3-Hydroxy-(4S,4aS,7S,7aS)-Dihydronepetalactone: 5.87%; (4aS,7S,7aS)-trans,cis-Nepetalactone: 4.3%; Linalool: 1.1%; (4aS,7S,7aR)-cis,trans-Nepetalactone: 1.07%; Linalyl acetate: 0.33%; Terpinen-4-ol: 0.27%	[30]
	4aα,7α,7aα-Nepetalactone: 64.9%; 4aα,7α,7aβ-Nepetalactone: 7.4%; α-Terpineol: 2.7%; 4aβ,7α,7aβ-Nepetalactone: 1.7%; α-Thujone: 0.5%; Verbenone: 0.3%	[16] Sample A
	4aβ,7α,7aβ-Nepetalactone: 33.6%; 4aα,7α,7aβ-Nepetalactone: 25.6%; 4aα,7α,7aα-Nepetalactone: 24.4%; 1,8-Cineole: 9%; Piperitone: 0.7%; α-Terpineol: 0.5%; Linalool: 0.4%; Terpinen-4-ol: 0.3%; δ-Terpineol: 0.2%	[16] Sample B
	4aα,7α,7aβ-Nepetalactone: 70%; 4aα,7α,7aα-Nepetalactone: 16.7%	[16] Sample C
**Sesquiterpene hydrocarbons**	Germacrene D: 7.39%; (E)-Caryophyllene: 2.09%; β-Elemene: 0.79%; β-Bourbonene: 0.67%; δ-Cadinene: 0.39%; α-Amorphene: 0.27%; α-Copaene: 0.24%; β-Cubebene: 0.23%; α-Farnesene: 0.23%; γ-Cadinene: 0.21%; α-Humulene: 0.13%	[26]
	(Z)-α-Bisabolene: 2.4%; β-Bourbonene: 0.6%; Germacrene D: 0.5%; β-Farnesene: 0.3%	[27]
	(E)-Caryophyllene: 3.5%; α-Muurolene: 1.4%; (E)-β-Farnesene:: 0.5%; γ-Cadinene: 0.5%; δ-Cadinene: 0.4%; β-Bourbonene: 0.1%	[28]
	β-Bourbonene: 3.3%; Germacrene D: 0.9%; Bicyclogermacrene: 0.2%; (E)-Caryophyllene: tr%	[29]
	Germacrene D: 4.7%; (E)-Caryophyllene: 3.7%; α-Copaene: 1.9%; δ-Cadinene: 1.5%; α-Humulene: 0.5%	[20]
	Germacrene D: 4.87%; (E)-Caryophyllene: 3.6%; α-Copaene: 1.93%; δ-Cadinene: 1.77%; α-Humulene: 0.67%	[30]
	Germacrene D: 0.6%; β-Farnesene: 0.3%	[16] Sample A
	γ-Muurolene: 0.6%; (E)-Caryophyllene: 0.4%; β-Farnesene: 0.2%	[16] Sample B
	Caryophyllene (unspecified): 0.3%;	[16] Sample C
**Oxygenated sesquiterpenes**	Caryophyllene oxide: 3.98%; 6-Isopropenyl-4,8a-dimethyl-1,2,3,5,6,7,8,8a-octahydronaphthalene-2-ol: 1.82%; Cycloisolongifolene, 8-hydroxy-, endo-: 1.13%; Salvial-4(14)-en-1-one: 0.93%; Spathulenol: 0.61%; β-Copaen-4α-ol: 0.5%; 1,5-Epoxysalvial-4(14)-ene: 0.39%; Widdrol: 0.33%	[26]
	Spathulenol: 1%; Caryophyllene oxide: 0.4%	[27]
	Spathulenol: 2.6%; α-Bisabolol: 0.2%	[28]
	Spathulenol: 0.2%	[29]
	Spathulenol: 0.3%	[20]
	Spathulenol: 0.6%	[30]
	(E)-Nerolidol: 8.8%; Spathulenol: 0.5%	[16] Sample A
**Others (non-terpenoids)**	n-Hexadecanoic acid: 6.47%; Phytol: 3.96%; Nonadecanoic acid: 2.82%; Methyl-11,14,17-eicosatrienoate: 1.46%; Hexadecanamide: 1.18%; Hexahydrofarnesyl acetone: 1.12%; Hexanedioic acid, bis(2-ethylhexyl) ester: 0.53%; 1-Tetradecanol: 0.51%; 1-Octadecanol: 0.34%; Tetradecanoic acid: 0.33%; Methyl linolenate: 0.27%; Heneicosane: 0.27%; (E)-2-Hexenal/2-Hexenal: 0.23%; Dodecanoic acid: 0.21%; Pentadecanoic acid, 14-methyl-, methyl ester: 0.2%; Nonanal: 0.18%; Hexadecanoic acid, butyl ester: 0.18%; 7,9-di-tert-butyl-1-oxaspiro[4.5]deca-6,9-diene-2,8-dione: 0.16%; n-Pentadecanoic acid: 0.14%; 1-Hexadecanol: 0.14%; Hexadecanal diallyl acetal: 0.12%; Trans,trans-2,4-Heptadienal: 0.1%; Octadecanal: 0.08%; 1-Octen-3-ol: 0.07%	[26]
	Manoyl oxide: 0.3%; (O)-Xylene: 0.1%	[27]
	(E)-2-Hexenal/2-Hexenal: 0.2%	[28]
	Styrene: 0.1%	[29]
	Other (unidentified) compounds: 5.3%; 3-Octanone: 0.3%	[20]
	Other (unidentified) compounds: 5.87%; 3-Octanone: 0.5%	[30]
	Unidentified compounds (6): 12.6%	[16] Sample C

Note: Compounds within each source are listed in decreasing order of relative abundance (%); tr = trace amount (<0.1%). Samples A–C correspond to three distinct NREO profiles compiled in the review by Salehi et al. [16]; Sample C was reported under the synonym *N. mussinii*.

**Table 3:** Antioxidant activity of DMEO and of EOs from other nepetalactone-rich *Nepeta* species.

Essential Oil	Assay	Result	Reference Standard	Reference
DMEO (250 mg/mL)	Lipid radical scavenging	397.20 ± 36.12 µmol TE/100 mL	Trolox (results expressed as TE)	[23]
DMEO (250 mg/mL)	ABTS•^+^ scavenging	312.54 ± 11.63 µmol TE/100 mL	Trolox (results expressed as TE)	[23]
DMEO (250 mg/mL)	Superoxide anion scavenging	294.77 ± 13.29 µmol TE/100 mL	Trolox (results expressed as TE)	[23]
DMEO (250 mg/mL)	DPPH• scavenging	246.39 ± 1.17 µmol TE/100 mL	Trolox (results expressed as TE)	[23]
DMEO (250 mg/mL)	Reducing power	171.46 ± 2.56 µmol TE/100 mL	Trolox (results expressed as TE)	[23]
DMEO, DMEO10%, DMNEO	DPPH and ABTS (IC_50_)	Potency ranking: BHT > DMEO > DMEO10% > DMNEO	BHT	[22]
DMEO (Egyptian-grown)	DPPH (IC_50_)	410 mg/mL	BHT: 3.18 mg/mL	[22]
*N. sintenisii* EO	DPPH (IC_50_)	7.16 mg/mL	–	[32,33]
*N. sintenisii* EO	FRAP	0.82 mM Fe^2+^/mg EO	–	[32,33]
*N. flavida* EO	DPPH (IC_50_)	42.8 ± 2.2 µg/mL	–	[15]
NREO	–	No data available for the EO	–	–

Note: TE: Trolox equivalents; BHT: butylated hydroxytoluene; IC_50_: concentration required for 50% inhibition; DMEO10%: 10% dilution of DMEO; DMNEO: Tween 80-based DMEO nano-emulsion; –: not reported. Data for *N. sintenisii* and *N. flavida* refer to *Nepeta* species other than *N. racemosa* and are presented for genus-level context only.

**Table 4:** Disk-diffusion inhibition zone diameters (mm) of DMEO.

Microorganism	Inhibition Zone—EO (mm)	Positive Control—Antibiotic (mm)	Reference
**Bacterial suspension: ~6 log CFU/mL (~1 × 10^6^ CFU/mL)**			
*S. aureus* ATCC 25923	40.0 mm	28.0 mm	[23]
*L. monocytogenes* ATCC 35152	40.0 mm	26.3 mm	
*E. coli* ATCC 25922	40.0 mm	27.0 mm	
*S.* Typhimurium ATCC 13311	40.0 mm	29.33 mm	
*P. aeruginosa* ATCC 27853	21.0 mm	21.0 mm	
*B. cereus* ATCC 11778	16.0 mm	27.0 mm	
**Bacterial suspension: 1.5 × 10^6^ CFU/mL**			
*E. coli*	10 mg/mL: 8.21 mm 15 mg/mL: 9.25 mm	Vancomycin: NG Ampicillin: 18.20 mm	[25]
*S.* Typhimurium	10 mg/mL: 8.55 mm 15 mg/mL: 9.27 mm	Vancomycin: NG Ampicillin: 18.80 mm	
*S. aureus*	10 mg/mL: 9.53 mm 15 mg/mL: 12.15 mm	Vancomycin: NG Ampicillin: NG	
*L. monocytogenes*	10 mg/mL: 9.11 mm 15 mg/mL: 11.21 mm	Vancomycin: 26.30 mm Ampicillin: 30.20 mm	
**Bacterial suspension: ~1.5 × 10^8^ CFU/mL**			
*S.* Typhimurium	DMEO: 59.67 mm DMEO10%: 14.67 mm DMNEO: 34.67 mm	Gentamicin: 21.33 mm Erythromycin: 11.00 mm Tetracycline: 17.33 mm Vancomycin: 17.33 mm Clindamycin: 20.00 mm	[22]
*E. coli*	DMEO: 60.00 mm DMEO10%: 13.67 mm DMNEO: 31.00 mm	Gentamicin: 22.67 mm Erythromycin: 11.33 mm Tetracycline: 18.67 mm Vancomycin: 18.00 mm Clindamycin: 21.33 mm	
*P. aeruginosa*	DMEO: 58.33 mm DMEO10%: 14.33 mm DMNEO: 30.00 mm	Gentamicin: 21.33 mm Erythromycin: 11.33 mm Tetracycline: 15.33 mm Vancomycin: 19.33 mm Clindamycin: 19.67 mm	
*Sh. dysenteriae*	DMEO: 51.67 mm DMEO10%: 13.00 mm DMNEO: 27.33 mm	Gentamicin: 23.00 mm Erythromycin: 11.00 mm Tetracycline: 16.33 mm Vancomycin: 18.33 mm Clindamycin: 20.33 mm	
*S. aureus*	DMEO: 62.00 mm DMEO10%: 16.00 mm DMNEO: 37.00 mm	Gentamicin: 21.33 mm Erythromycin: 14.33 mm Tetracycline: 21.00 mm Vancomycin: 20.67 mm Clindamycin: 21.33 mm	
*B. cereus*	DMEO: 70.33 mm DMEO10%: 16.33 mm DMNEO: 42.00 mm	Gentamicin: 22.00 mm Erythromycin: 12.33 mm Tetracycline: 19.33 mm Vancomycin: 20.67 mm Clindamycin: 21.67 mm	
*L. monocytogenes*	DMEO: 61.67 mm DMEO10%: 13.67 mm DMNEO: 29.10 mm	Gentamicin: 22.33 mm Erythromycin: 13.67 mm Tetracycline: 18.00 mm Vancomycin: 19.67 mm Clindamycin: 24.67 mm	

Note: DMEO: neat essential oil; DMEO10%: 10% dilution of DMEO; DMNEO: Tween 80-based DMEO nano-emulsion; NG: no growth.

**Table 5:** Minimum inhibitory concentration (MIC) and minimum bactericidal concentration (MBC) of DMEO.

Microorganism	MIC	MBC/MFC	Reference
**Bacterial suspension ~1.5 × 10^5^ CFU/mL**			
*E. coli* PTCC 1533	2.00 mg/mL	4.00 mg/mL	[25]
*S.* Typhimurium PTCC 1730	2.00 mg/mL	4.00 mg/mL	
*S. aureus* PTCC 1015	0.12 mg/mL	0.25 mg/mL	
*L. monocytogenes* PTCC 1298	1.00 mg/mL	4.00 mg/mL	
**Neat (DMEO), 10% dilution (DMEO10%) and Tween-80 nano-emulsion (DMNEO)**			
*S.* Typhimurium ATCC 14028	DMEO: 2.5 mg/mL DMEO10%: 64 mg/mL DMNEO: 4 mg/mL	DMEO: 5 mg/mL DMEO10%: 128 mg/mL DMNEO: 8 mg/mL	[22]
*E. coli* ATCC 25922	DMEO: 1.25 mg/mL DMEO10%: 64 mg/mL DMNEO: 8 mg/mL	DMEO: 5 mg/mL DMEO10%: 128 mg/mL DMNEO: 32 mg/mL	
*P. aeruginosa* ATCC 27853	DMEO: 1.25 mg/mL DMEO10%: 128 mg/mL DMNEO: 8 mg/mL	DMEO: 2.5 mg/mL DMEO10%: 256 mg/mL DMNEO: 32 mg/mL	
*Sh. dysenteriae* PTCC 188	DMEO: 0.625 mg/mL DMEO10%: 128 mg/mL DMNEO: 4 mg/mL	DMEO: 5 mg/mL DMEO10%: 128 mg/mL DMNEO: 8 mg/mL	
*S. aureus* ATCC 25923	DMEO: 0.312 mg/mL DMEO10%: 32 mg/mL DMNEO: 8 mg/mL	DMEO: 2.5 mg/mL DMEO10%: 64 mg/mL DMNEO: 16 mg/mL	
*B. cereus* ATCC 14579	DMEO: 1.25 mg/mL DMEO10%: 32 mg/mL DMNEO: 8 mg/mL	DMEO: 2.5 mg/mL DMEO10%: 64 mg/mL DMNEO: 16 mg/mL	
*L. monocytogenes* ATCC 19115	DMEO: 1.25 mg/mL DMEO10%: 64 mg/mL DMNEO: 8 mg/mL	DMEO: 2.5 mg/mL DMEO10%: 256 mg/mL DMNEO: 64 mg/mL	

Note: DMEO: neat essential oil; DMEO10%: 10% dilution of DMEO; DMNEO: Tween 80-based DMEO nano-emulsion.

**Table 6:** Minimum inhibitory concentration (MIC) and minimum bactericidal concentration (MBC) of *Nepeta* species.

Microorganism	Species	MIC	MBC/MFC	Reference
*E. coli* (cephalosporin-sensitive)	*N. cataria* EO	3.17–4.00 µL/mL	n.r.	[16]
*S. aureus*	*N. cataria* EO	0.15–0.22 µL/mL	n.r.	
*S. aureus*	*N. ucrainica* ssp. *kopetdaghensis* EO	14 µg/mL	14 µg/mL	
*M. luteus*	*N. ucrainica* ssp. *kopetdaghensis* EO	28 µg/mL	55 µg/mL	
*P. aeruginosa*/*E. coli*	*N. ucrainica* ssp. *kopetdaghensis* EO	>150 µg/mL	>150 µg/mL	
*S. aureus*/*K. pneumoniae*/*E. coli*	*N. sessilifolia* EO	125–500 mg/mL	n.r.	[33]
Most sensitive plant/food-borne pathogens	*N. nuda* EO	7.81 mg/mL	n.r.	

Note: ssp.: subspecies; n.r.: not reported. Data refer to *Nepeta* species other than *N. racemosa* and are presented for genus-level context only.

**Table 8:** Summary of key toxicological data for the ten principal DMEO and NREO constituents.

Compound	CAS No.	Oral LD50 (Rodent)	Key GHS Hazards	Genotoxicity/Carcinogenicity	Regulatory Status
***DMEO***					
Geranyl acetate	105-87-3	6330 mg/kg (rat, oral)	Skin Irrit. 2 (H315); Skin Sens. 1 (H317)	Ames negative; positive in mouse lymphoma and some chromosomal aberration assays; not carcinogenic (NTP)	JECFA ADI 0–0.5 mg/kg bw; FDA GRAS (21 CFR 182.60)
Geranial/Citral	141-27-5/5392-40-5	≈1440–6000 mg/kg	Skin Irrit. 2 (H315); Skin Sens. 1 (H317); Eye Irrit. 2 (H319, ≈85%)	Negative across Ames, CA, and micronucleus tests; equivocal carcinogenicity in female mice (NTP)	EFSA group ADI 0.05 mg/kg bw/day; FDA GRAS (21 CFR 182.60)
Neral	106-26-3	≈4895–6000 mg/kg	Skin Irrit. 2 (H315); Skin Sens. 1B (H317)	Negative (EFSA); not listed as carcinogen (IARC)	Evaluated by EFSA
Geraniol	106-24-1	3600 mg/kg (rat, oral)	Skin Irrit. 2 (H315); Skin Sens. 1 (H317); Eye Dam. 1 (H318)	Inconsistent across EFSA assessment years; RIFM 2022: not genotoxic	FDA GRAS (21 CFR 182.60)
Linalool	78-70-6	2790 mg/kg (rat, oral)	Skin Irrit. 2 (H315); Skin Sens. 1B (H317); Eye Irrit. 2 (H319)	Negative across full battery (EFSA 2020)	JECFA ADI 0–0.5 mg/kg bw; FDA GRAS (21 CFR 182.60)
***NREO***					
Nepetalic acid	524-06-1	No data identified	No data identified	No data identified	No data identified
Nepetalactone	490-10-8	1550 mg/kg (mouse, i.p.)	Not classified (single ECHA industry report)	Not determined	No data identified
Germacrene D	23986-74-5	No quantitative value identified	≈60% H302/H304; ≈40% not classified	Not determined (EFSA FGE.25, 2008/2010/2011)	Evaluated by EFSA FGE.25
1,8-cineole	470-82-6	2480–4700 mg/kg (rat, oral)	Flam. Liq. 3 (H226); Skin Sens. 1 (H317)	Negative (EFSA 2008, 2011); no evidence of carcinogenicity (80-week mouse study)	JECFA safe as flavoring; FDA (21 CFR 172.515)
Palmitic acid	57-10-3	57 mg/kg (mouse, i.v.)	Mostly not classified (66.5%); minority H315/H319/H335	No indication of carcinogenicity (IARC)	CIR safe; FDA (21 CFR 172.860); EPA Safer Choice

Note: Cells marked “no data identified” reflect a genuine absence of published toxicological data in PubChem and associated regulatory databases (ECHA, EFSA), not an omission from this review. All data refer to isolated constituents and derive mainly from regulatory assessments and toxicological databases; no corresponding data are available for DMEO or NREO as whole oils.

**Table 9:** Evidence on anti-*Helicobacter pylori* activity relevant to DMEO and NREO, grouped by level of evidence.

Level of Evidence	EO/Compound	Main Finding	Type of Study	Reference
DMEO	DMEO	No direct data available	–	–
NREO	NREO	No direct data available	–	–
Other *Nepeta* spp.	*N. argolica* subsp. *dirphya* EO (nepetalactone-rich); *N. camphorata* EO	Inhibition of *H. pylori* growth (MIC 128 µg/mL)	In vitro	[31,73]
Individual DMEO constituents	Citral (geranial + neral)	Among the most potent anti-*H. pylori* constituents; MBC 40 µg/mL	In vitro	[71,73]
	Geraniol	MBC 100 µg/mL; anti-ulcer effect and 50% reduction in infection, alone or with clarithromycin	In vitro; in vivo (rats)	[73]
Individual NREO constituents	Nepetalactone stereoisomers	MIC/MBC 64 and 128 µg/mL	In vitro	[73]
	1,8-Cineole	Essentially inactive against clinical isolates	In vitro	[74]
Unrelated EOs	Lemongrass EO (*Cymbopogon citratus*, citral-rich)	Among the most active EOs against the reference strain; more moderate activity against clinical isolates	In vitro	[76]
	*Salvia officinalis* EO (1,8-cineole-rich)	Moderate activity	In vitro	[77]

Note: MIC: minimum inhibitory concentration; MBC: minimum bactericidal concentration; –: not applicable.

## Data Availability

No new data were created or analyzed in this study. Data sharing is not applicable to this article.
